# Synthesis, Antibacterial and Anthelmintic Activity of Novel 3-(3-Pyridyl)-oxazolidinone-5-methyl Ester Derivatives

**DOI:** 10.3390/molecules27031103

**Published:** 2022-02-07

**Authors:** Bo Jin, Jia-yi Chen, Zun-lai Sheng, Meng-qing Sun, Hong-liang Yang

**Affiliations:** 1 Department of Veterinary Medicine, Northeast Agricultural University, Harbin 150030, China; cnborgin@163.com (B.J.); bear2320581078@163.com (J.-y.C.); shengzunlai@neau.edu.cn (Z.-l.S.); smq1536578652@163.com (M.-q.S.); 2 Heilongjiang Key Laboratory for Animal Disease Control and Pharmaceutical Development, Northeast Agricultural University, Harbin 150030, China

**Keywords:** pyridinyl-oxazolidinone derivatives, synthesis, antibacterial activity, molecular docking, anthelmintic activity

## Abstract

In this study, a series of 3-(3-pyridyl)-oxazolidone-5-methyl ester derivatives was synthesized and characterized by ^1^H NMR, ^13^C NMR, and LC-MS. The conducted screening antibacterial studies of the new 3-(3-pyridyl)-oxazolidone-5-methyl ester derivatives established that the methyl sulfonic acid esters have broad activity spectrum towards *Staphylococcus aureus*, *Streptococcus pneumoniae*, *Bacillus subtilis* and *Staphylococcus epidermidis.* Among them, compound **12e** has the most potent activity, with an MIC of 16 μg/mL against *B.subtilis,* and could reduce the instantaneous growth rate of bacteria. Furthermore, molecular docking studies were also simulated for compound **12e** to predict the specific binding mode of this compound. In addition, anthelmintic activity of these compounds was also evaluated against adult Indian earthworms (*Pheretima posthuman*). The results showed that compound **11b** had the best effect. These results above can provide experimental reference for the development of novel antibacterial and anthelmintic drugs.

## 1. Introduction

With bacterial resistance comes a grave threat to global public health security. Owing to the rapid growth of clinical drug-resistant bacteria, the number of effective anti-infective drugs has declined, and patient mortality is increasing [1,2,3,4,5]. It is estimated that about 10 million people all over the world will die from drug-resistant bacterial infections, which will become the leading cause of human disease death, and lead to the loss of global GDP of US $100 trillion by 2050 [6]. Therefore, how to effectively deal with the bacterial resistance crisis is a challenging question. Many new therapies, such as those utilizing nanoparticles, phages, and protic ionic liquids, are being developed [7,8,9,10,11]. However, these technologies are still immature and have not been effectively used clinically [12,13,14]. At present, chemosynthesized antibacterial agents still play an indispensable role in clinical practice. Therefore, for pharmaceutical chemists all over the world, the discovery of novel active antibacterial compounds is an urgent mission and the direction of continuous efforts.

Linezolid, an oxazolidinone antibacterial agent, has been widely used in the treatment of infection caused by multi-drug-resistant Gram-positive bacteria since it was approved for clinical use in 2000. However, with clinical use gradually increasing, many linezolid-resistant strains have also been reported [15,16,17,18]. To find novel, safe and effective antibacterial drugs, there have been many studies in recent years on the structural modification of linezolid [19,20,21,22,23,24,25]. Among them, many have focused on the modification of five-position side chains of oxazolidine and the morpholine ring, while a few have explored the benzene ring of linezolid. In addition, anthelmintic properties of linezolid derivatives have also been found [26].

**Figure 1:**
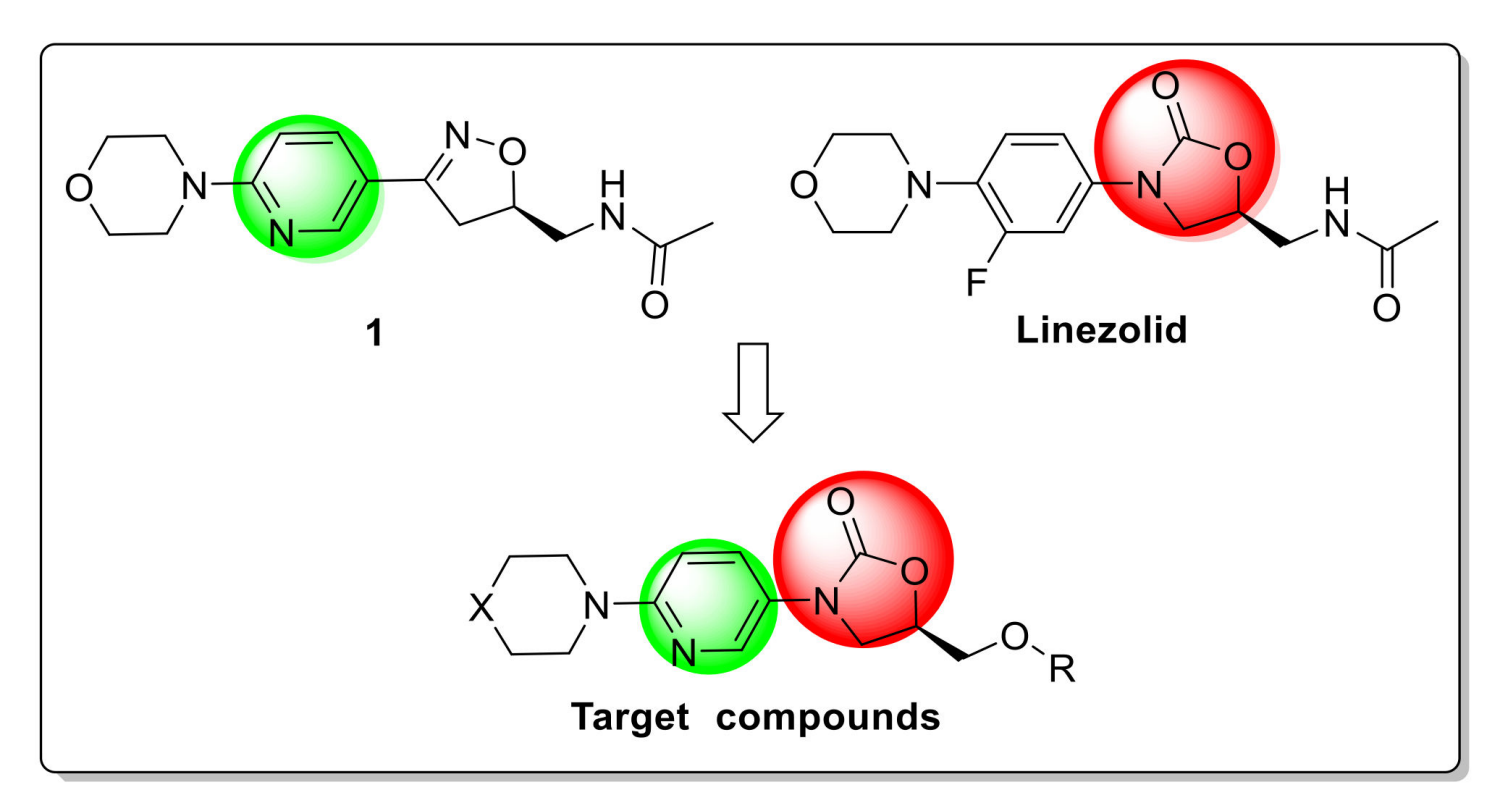
Design strategy of 3-(3-pyridyl)-oxazolidone-5-methyl ester derivatives.

The pyridine heterocyclic ring has been widely used in the medicine field because of its distinctive aromatic and electronegative properties [27,28,29,30,31]. In our previous studies, a series of pyridine heterocyclic derivatives were evaluated to have good biological activities [32,33,34]. Among them, compound **1** showed good antibacterial and anthelmintic activities in vitro. Based on previous research and theory [35], here we replaced the benzene ring with pyridine and a nitrogen atom with an oxygen atom at the 5-position side chain of the oxazolidone ring. A series of 3-(3-pyridyl)-oxazolidinone-5-methyl ester derivatives were designed and synthesized (Figure 1), and their antibacterial and anthelmintic effects in vitro were evaluated.

## 2. Results and Discussion

### 2.1. Chemistry

**Scheme 1:**
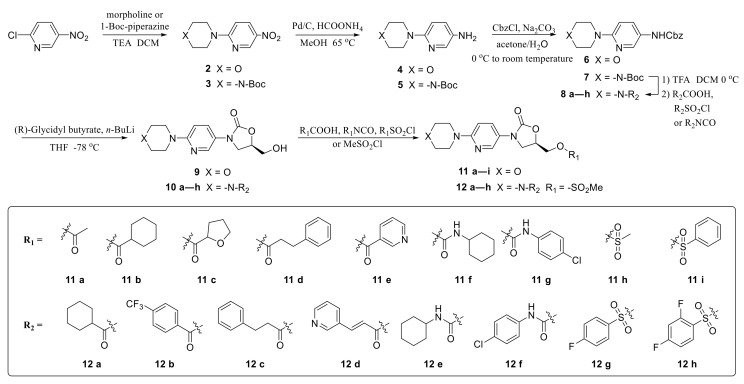
Synthesis route of the target compounds.

The general synthetic route for the target compounds is illustrated in Scheme 1. The commercially available 2-chloro-5-nitropyridine was reacted with morpholine to produce intermediate **2**. Then, the nitro group was reduced to prepare amine **4**. After the introduction of the Cbz group, intermediate **6** was cyclized with *(R)*-(-)-butylglycidyl ester under the condition of −78 °C and n-butyllithium (n-BuLi) to produce oxazolidinone intermediate **9**. Finally, ester derivatives **11a–i** were prepared with intermediate **9** and different acids, sulfonyl chlorides and isocyanates. Compounds **12a–h** were obtained by similar methods.

### 2.2. Antibacterial Activity Assay

#### 2.2.1. Screening of Antibacterial Activity

All twelve synthesized compounds were tested against four Gram-positive strains, including *S. aureus* (ATCC25923), *S. pneumoniae* (ATCC49619), *B. subtilis* (BNCC109047) and *S. epidermidis* (BNCC186652) by broth dilution method with linezolid as the standard drug, and the MIC values are listed in Table 1.

Initially, keeping the morpholine ring of linezolid in our structure, compounds **11a–i** were synthesized and evaluated for antibacterial activity against four gram-positive strains. Unfortunately, the activity of most compounds was generally weak, with only compounds **11f** and **11h** showing a broad-spectrum effect.

**Figure 2:**
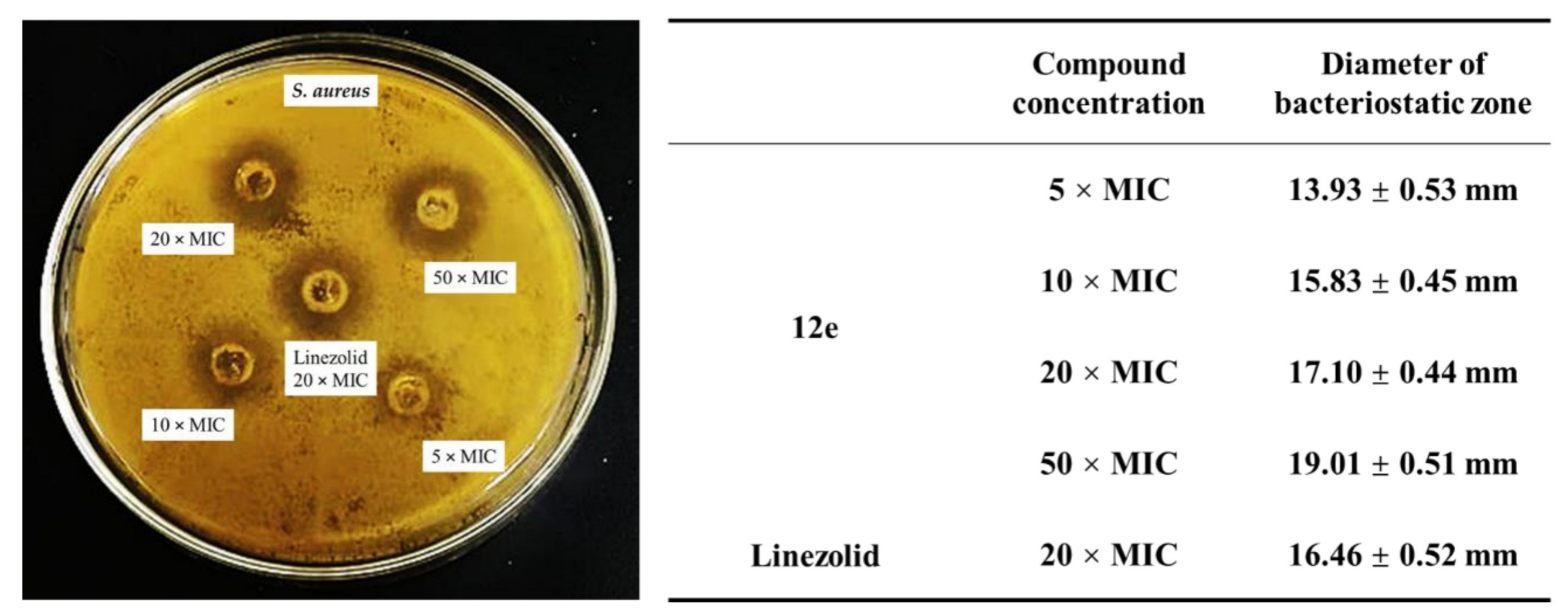
Bacteriostatic zone of **12e** against *S. aureus* at different concentrations. (MIC: minimal inhibit concentration).

Therefore, while maintaining the methyl sulfonyl group on the C-5 side chain, compounds **12a–h** were synthesized by replacing the morpholine ring with a piperazine ring and modifying the side chain further. Their activity was significantly improved and they all had broad-spectrum antibacterial action. Among these compounds, compounds **12a** and **12e** had the best antibacterial activity, with the lowest MIC values reaching 16 μg/mL, indicating that the flexible substituents linked with piperazine had stronger antibacterial activity. The activity of **12c** and **12d** was slightly weaker than that of **12a** and **12e**, probably due to the presence of long-chain substituents, which were less binding than flexible rings, but their long chains were morphologically consistent with the target. Conversely, rigid rings, such as **12b**, reduced antibacterial activity. Compared with our previous work, these compounds still retain antibacterial activity after changing the core structure to oxazolidinone [34]. Here the antibacterial activity of the synthesized compounds was not as good as linezolid, which may be due to its reduced affinity with the target caused by the structural changes. In addition, a bacterial inhibitory zone test of **12e** against *S. aureus* was provided to visually observe bacteriostatic effects of **12e** (Figure 2). It can be seen from the figure that the bacteriostatic zone increased with the increase of drug concentration, and the diameter of the zone was similar to that of linezolid at a concentration of 20 × MIC.

#### 2.2.2. Bacterial Growth Kinetic Study

**Figure 3:**
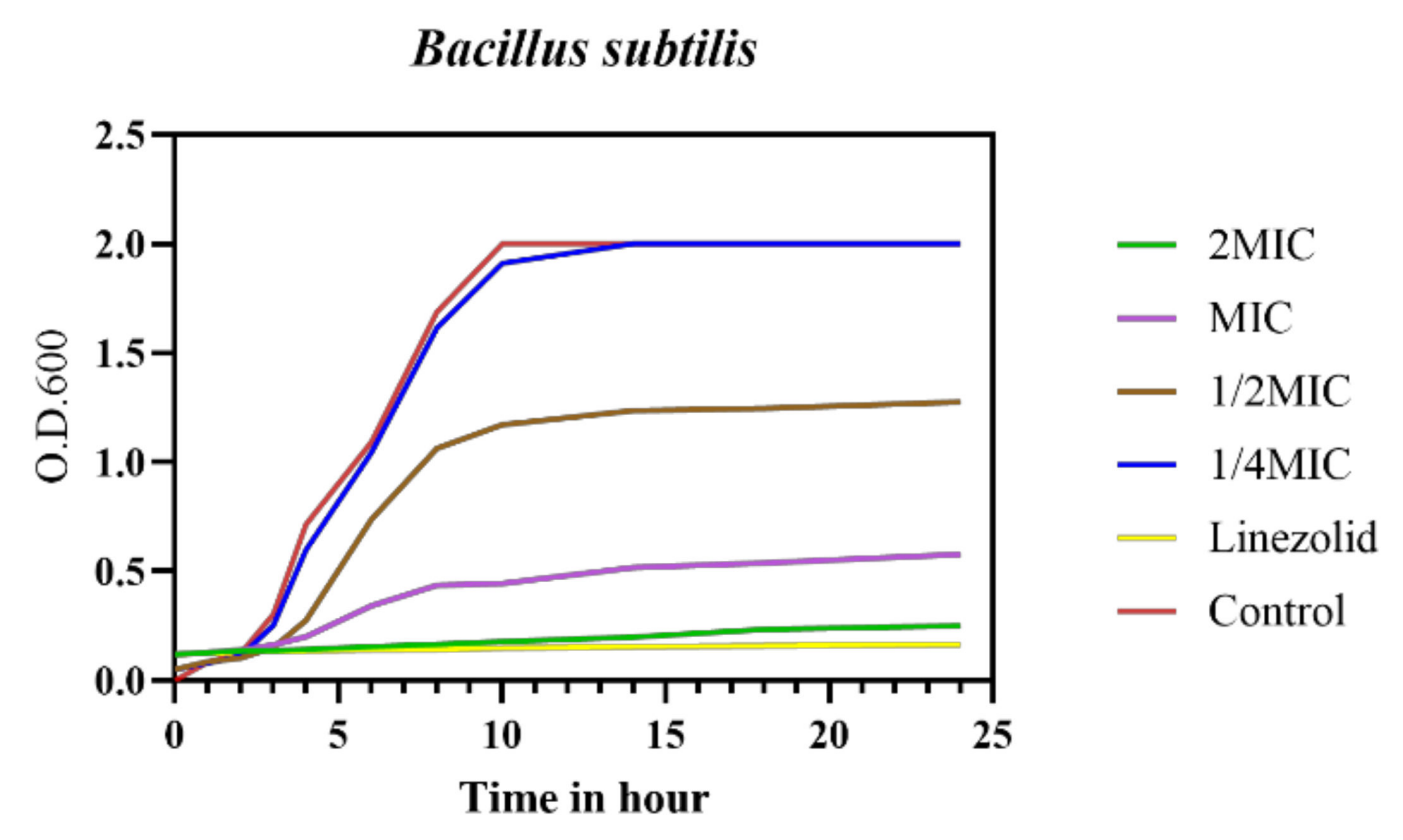
Inhibition of the growth of Bacillus subtilis by different concentrations of compound **12e**. (MIC: minimal inhibit concentration; O. D.: optical density.)

The growth curve of bacteria directly shows the process and rule of the change of bacterial concentration with time, while the growth inhibition curve of bacteria can directly reflect the influence of drugs on the growth process of bacteria [36]. The growth inhibition curve of compound **12e** against *Bacillus subtilis* is shown in Figure 3.

Without treatment (red) or the presence of 1/4 MIC (blue) of **12e**, the bacteria increased sharply in the log phase (2–10 h). With the increase in concentration, the lag phase was prolonged and the duration of the logarithmic phase was shortened. The significantly change was that the decreased instantaneous growth rate led to the plateau phase concentration reduction, which was manifested by the difference of the final turbidity of bacterial liquid. Until 2 × MIC of **12e** (green) and linezolid (yellow), there was no obvious growth of bacteria.

These findings revealed that **12e** was an excellent bactericidal agent in a concentration-dependent manner, and mainly disturbed the instantaneous growth rate of bacteria in the logarithmic phase.

### 2.3. Binding Mode Study

To explore the mechanism of the active compound **12e**, Auto-Dock software was used to perform docking analysis with the 50S ribosomal subunit (PDB ID: 3CPW).

**Figure 4:**
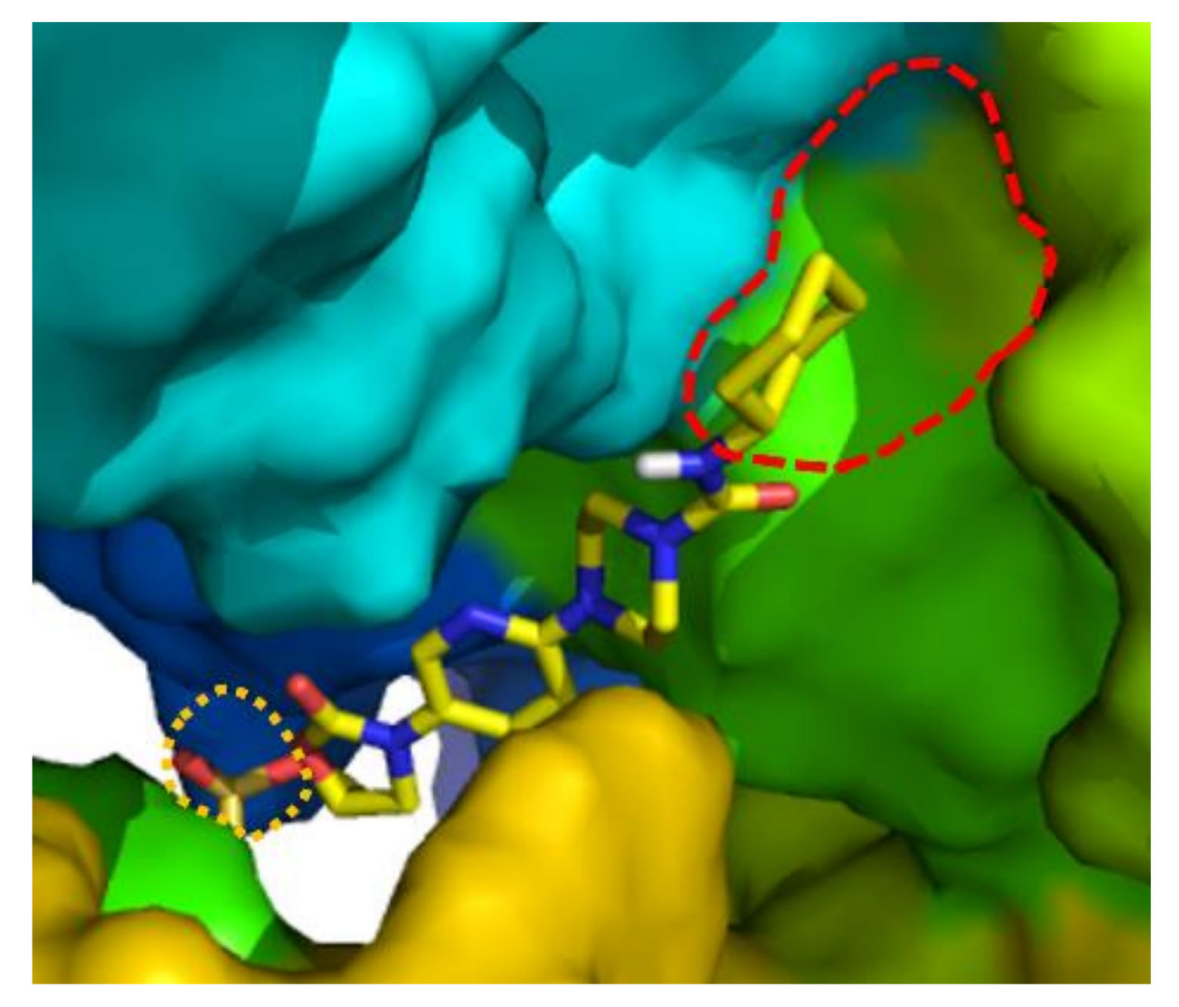
Binding pocket of compound 12b with the 50S ribosomal subunit.

The docking result from Figure 4 demonstrated that **12e** was inclined to locate in a long and narrow channel with a fully extended state. The five-position side chain of the oxazolidine ring was binding to a relatively small cavity (yellow), which could only accommodate a small group. This might be the reason for the moderate activity of compound **11h**. In addition, it was discovered that the 4′-position side chain of the piperazine ring extended into a deep cavity (red), which could accommodate bigger structural groups after containing the flexible cyclohexyl group. Presumably, compounds **12a–h** had better antibacterial effect than **11a–i**, because they were suitable for the narrow cavity (yellow), and occupied the larger and deeper region (red) at the same time, making them more closely combined with the target.

**Figure 5:**
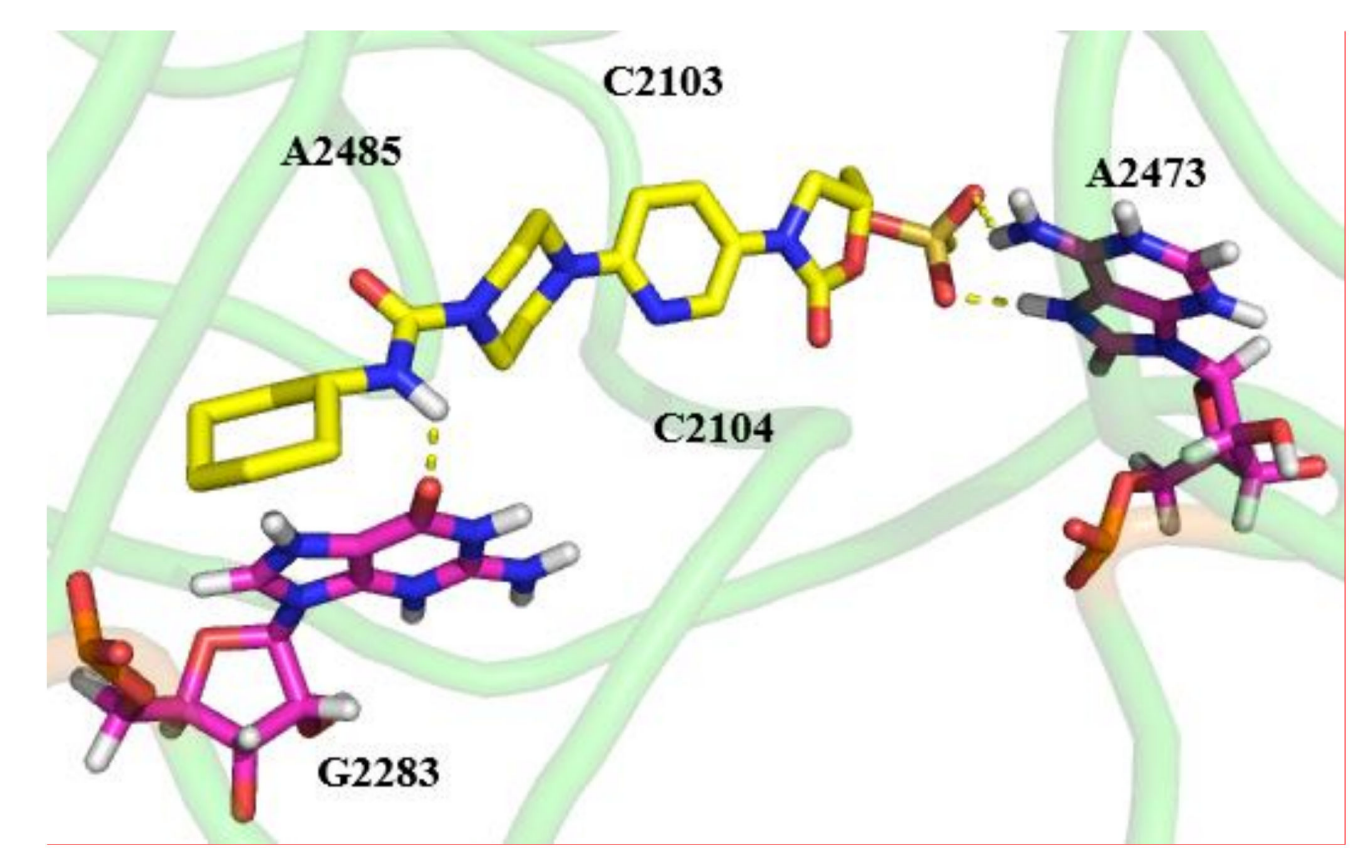
Binding mode of compound **12e** to the ribosomal subunit 50S.

The specific mechanism of action is shown in Figure 5; the oxygen atoms of the sulfonyl group formed two hydrogen bonds with A2473. It followed that the sulfonyl group presumedly had better ability to form hydrogen bonds than the carbonyl group, which explained why **11h** had the best antibacterial activity among **11a–i**. In addition, the piperazine-linked urea group of **12e** extended into the deep cavity forming a hydrogen bond with G2283, making **12b** more effective.

### 2.4. Anthelmintic Activity Assay

Anthelmintic activity assay of 17 target compounds was performed using adult Indian earthworm (*Pheretima posthuman*) with albendazole as the positive standard drug. Efficacy was assessed by counting the paralysis and death time, and the results are shown in Table 2.

For compounds **11a–i**, the paralysis time of most compounds was less than 15 min, and the death time was more than 50 min, indicating that these compounds had significant paralysis effect on earthworms, but poor insecticidal action. In terms of shorter paralysis time (3.6 ± 0.2 min) and death time (8.1 ± 0.8 min), the anthelmintic effect of compound **11b** was better than albendazole and our previous compounds, indicating that it has potential to be developed into a new anthelmintic drug [34]. For compounds **12a–h**, the paralysis time was more than 15 min and death time was not discovered within 50 min, indicating that these compounds had poor anthelmintic activity, even though they had good antibacterial activity.

## 3. Materials and Methods

### 3.1. Chemical Reagents and Instruments

Before using, tetrahydrofuran (THF) was dried with sodium under reflux, dichloromethane (DCM) was dried with CaH_2_ under reflux and triethylamine (TEA) was dried with molecular sieve. All the reagents above were chemically pure (Tianjin Tianli Chemical Reagent Co., Ltd., Tianjin, China). All the other reagents and compounds used in this study were commercially available analytically pure (AR) without further purification. 

All the chemical reactions were detected by thin-layer chromatography (TLC), which was conducted on silica gel G plates (Taizhou Luqiao Sijia Biochemical Plastic Products Factory, Taizhou, China). The plates were observed under a Zf-2 tri-use ultraviolet analyzer (Shanghai Anting Electronic Instruments Factory, Shanghai, China). Most of the reaction mixtures were purified by column chromatography carried out on 200–300 mesh sieve specifications Silica Gel (Qingdao Ocean Chemical Co., Ltd., Qingdao, China).

All the purified compounds underwent the following tests to verify their purity and structure: a WRS-1A melting point apparatus (Shanghai Shinuo Physical Optical Instrument Co., Ltd., Shanghai, China) was used to assess the compounds’ melting points. The ^1^H-NMR and ^13^C-NMR of each compound were recorded on an NMR spectrometer (Varian, Salt Lake City, UT, USA) in DMSO-*d_6_* with tetramethyl-silane (TMS) as an internal standard. The mass spectra (MS) of each compound were registered in an Agilent 1100 liquid chromatography-mass spectrometry (LC/MS) instrument (Agilent Technologies Co., Ltd., Santa Clara, CA, USA).

In addition, all the work in the biological activity test was completed in a DL-CJ-2N high-performance aseptic test stand (Beijing Donglian Haer Instrument Manufacturing Co., Ltd., Beijing, China). All biological media such as Mueller–Hinton (MH) broth medium, Luria–Bertani (LB) broth medium and Nutrient Agar (Qingdao High-tech Park Haibo Biotechnology Co., Ltd., Qingdao, China) were all dissolved directly with deionized water then sterilized by a vertical autoclave (Zealway Instrument Co., Ltd., Xiamen, China). Bacteria used in this experiment were grown in an MCO-20AIC carbon dioxide incubator (SANYO Co., Ltd., Osaka, Japan).

### 3.2. Synthesis of the Target Compounds

Intermediates **2–10** were synthesized according to the steps in the literature [31,32,33,34].

#### 3.2.1. General Procedure for the Preparation of Compounds (**11a–e**)

Take compound *(R)*-(3-(6-morpholinopyridin-3-yl)-2-oxooxazolidin-5-yl) methyl acetate (**11a**) as an example. To a solution of compound **9** (0.29 mmol), 4-dimethyl aminopyridine (DMAP, 0.06 mmol) and 1-ethyl-3-(3-dimethyl aminopropyl) carbodiimide (EDCI, 1.5 mmol) in 4 mL DCM, was added drop of acetyl chloride (0.38 mmol) and stirred overnight. After the reaction completed, the reaction mixture was extracted with water and DCM (5 mL × 3). The combined organic phase was dried with anhydrous Na_2_SO_4_ and concentrated under reduced pressure. The target compound (**11a**) was separated by column chromatography (PE/EA, 3/1). Yield 72%. A white solid, m. p. 92.9–95.1 °C; ^1^H NMR (600 MHz, DMSO-*d_6_*) *δ* 8.23 (d, *J* = 3.0 Hz, 1H, H_2_-pyridine), 7.83 (dd, *J* = 9.0, 3.0 Hz, 1H, H_4_-pyridine), 6.89 (d, *J* = 9.0 Hz, 1H, H_5_-pyridine), 4.94–4.90 (m, 1H, H_5_-oxazolidone), 4.31–4.24 (m, 2H, OCH_2_), 4.12–4.10 (m, 1H, H_4_-oxazolidone), 3.80–3.77 (m, 1H, H_4_-oxazolidone), 3.72–3.68 (m, 4H, morpholine), 3.42–3.37 (m, 4H, morpholine), 2.06 (s, 3H, CH_3_); ^13^C NMR (150 MHz, DMSO-*d_6_*) *δ* 170.7, 156.8, 154.9, 139.2, 130.0, 126.8, 107.5, 71.1, 66.4, 64.8, 47.0, 46.0, 21.0; ES-MS: *m/z* calcd. for C_15_H_19_N_3_O_5_ [M + H]^+^: 322.1; found: 322.110.

*(R)-(3-(6-morpholinopyridin-3-yl)-2-oxooxazolidin-5-yl)methyl cyclohexanecarboxylate* (**11b**).

A pink solid; yield 65%; m. p. 96.6–99.1 °C; ^1^H NMR (600 MHz, DMSO-*d*_6_) *δ* 8.24 (d, *J* = 3.0 Hz, 1H, H_2_-pyridine), 7.83 (dd, *J* = 9.0, 3.0 Hz, 1H, H_4_-pyridine), 6.90 (d, *J* = 9.0 Hz, 1H, H_5_-pyridine), 5.03–4.96 (m, 1H, H_5_-oxazolidone), 4.23–4.18 (m, 1H, OCH_2_), 4.04–3.92 (m, 2H, OCH_2_ and H_4_-oxazolidone), 3.76–3.74 (m, 1H, H_4_-oxazolidone), 3.72–3.68 (m, 4H, morpholine), 3.42–3.38 (m, 4H, morpholine), 3.09–3.05 (m, 4H, cyclohexane), 1.20–1.18 (m, 7H, cyclohexane); ^13^C NMR (150 MHz, DMSO-*d*_6_) *δ* 175.8, 159.3, 154.4, 146.4, 135.1, 114.8, 106.7, 78.9, 65.8, 44.8, 43.8, 41.5, 28.6, 25.3, 25.2, 14.1; ES-MS: *m/z* calcd. for C_20_H_27_N_3_O_5_ [M + H]^+^: 390.2; found: 390.204.

*((R)-3-(6-morpholinopyridin-3-yl)-2-oxooxazolidin-5-yl)methyl tetrahydrofuran-2-carboxylate* (**11c**).

Red oil; yield 51%; ^1^H NMR (600 MHz, DMSO-*d*_6_) *δ* 8.23 (d, *J* = 3.0 Hz, 1H, H_2_-pyridine), 7.83 (dd, *J* = 9.0, 3.0 Hz, 1H, H_4_-pyridine), 6.90 (d, *J* = 9.0 Hz, 1H, H_5_-pyridine), 4.97–4.93 (m, 1H, H_5_-oxazolidone), 4.48–4.27 (m, 3H, tetrahydrofuran), 4.15–4.12 (m, 1H, OCH_2_), 3.82–3.73 (m, 3H, OCH_2_ and H_4_-oxazolidone), 3.73–3.66 (m, 4H, morpholine), 3.44–3.38 (m, 4H, morpholine), 2.17–2.11 (m, 1H, tetrahydrofuran), 1.93–1.70 (m, 3H, tetrahydrofuran); ^13^C NMR (150 MHz, DMSO-*d*_6_) *δ* 173.1, 159.4, 154.4, 146.4, 135.1, 114.7, 106.7, 78.5, 77.6, 68.5, 65.8, 44.8, 41.4, 37.2, 30.0, 24.8; ES-MS: *m/z* calcd. for C_18_H_23_N_3_O_6_ [M + H]^+^: 378.2; found: 378.138.

*(R)-(3-(6-morpholinopyridin-3-yl)-2-oxooxazolidin-5-yl)methyl benzoate* (**11d**).

A red solid; yield 62%; m. p. 97.4–100.0 °C; ^1^H NMR (600 MHz, DMSO-*d*_6_) *δ* 8.24 (d, *J* = 3.0 Hz, 1H, H_2_-pyridine), 7.83 (dd, *J* = 9.0, 3.0 Hz, 1H, H_4_-pyridine), 7.30–7.12 (m, 5H, benzene), 6.89 (d, *J* = 9.0 Hz, 1H, H_5_-pyridine), 4.92–4.89 (m, 1H, H_5_-oxazolidone), 4.35–4.24 (m, 2H, OCH_2_), 4.13–4.08 (m, 1H, H_4_-oxazolidone), 3.79–3.72 (m, 1H, H_4_-oxazolidone), 3.72–3.67 (m, 4H, morpholine), 3.45–3.34 (m, 4H, morpholine), 2.85–2.82 (m, 2H, CH_2_), 2.68–2.64 (m, 2H, CH_2_); ^13^C NMR (150 MHz, DMSO-*d*_6_) *δ* 171.9, 159.4, 154.5, 146.4, 141.2, 135.2, 128.2, 128.1, 125.8, 114.8, 106.7, 78.9, 65.9, 44.8, 42.0, 41.6, 36.8, 31.1; ES-MS: *m/_Z_* calcd. for C_22_H_25_N_3_O_5_ [M + H]^+^: 412.2; found: 412.148.

*(R)-(3-(6-morpholinopyridin-3-yl)-2-oxooxazolidin-5-yl)methyl nicotinate* (**11e**).

A red solid; yield 65%; m. p. 120.3–121.0 °C; ^1^H NMR (600 MHz, DMSO-*d*_6_) *δ* 9.05 (d, *J* = 1.8 Hz, 1H, pyridine), 8.82 (dd, *J* = 4.8, 1.8 Hz, 1H, pyridine), 8.29–8.23 (m, 2H, pyridine (B ring) and pyridine), 7.84 (dd, *J* = 9.0, 3.0 Hz, 1H, H_4_-pyridine (B ring)), 7.57 (dd, *J* = 7.8, 4.8 Hz, 1H, pyridine), 6.90 (d, *J* = 9.0 Hz, 1H, H_5_-pyridine (B ring)), 5.13–5.06 (m, 1H, H_5_-oxazolidone), 4.66–4.53 (m, 2H, OCH_2_), 4.23–4.22 (m, 1H, H_4_-oxazolidone), 4.01–3.99 (m, 1H, H_4_-oxazolidone), 3.72–3.68 (m, 4H, morpholine), 3.42–3.38 (m, 4H, morpholine); ^13^C NMR (150 MHz, DMSO-*d*_6_) *δ* 165.3, 159.4, 154.5, 151.9, 148.4, 146.5, 135.2, 135.0, 129.7, 123.4, 114.7, 106.7, 78.5, 65.9, 44.8, 42.6, 37.5; ES-MS: *m/z* calcd. for C_19_H_20_N_4_O_5_ [M + H]^+^: 385.1; found: 385.133.

Raw data for the above products are presented in Supplementary Materials (Figures S1–S16).

#### 3.2.2. General Procedure for the Preparation of Compounds (**11f**–**i** and **12a**–**h**)

Take compound *(R)*-(3-(6-morpholinopyridin-3-yl)-2-oxooxazolidin-5-yl) methyl cyclohexylcarbamate (**11f**) as an example. To a solution of compound **9** (0.29 mmol), TEA (0.58 mmol) in 4 mL DCM was added drop of isocyanatocyclohexane (0.44 mmol) under 0 °C, then the mixture was raised to room temperature and stirred overnight. After the reaction completed, the mixture was extracted with water and DCM (5 mL × 3). The combined organic phase was dried with anhydrous Na_2_SO_4_ and concentrated under reduced pressure. The mixture was recrystallized with petroleum ether (PE) and ethyl acetate (EA) to obtain compound (**11f**). Yield 65%. A red solid, m. p. 200.7–203.0 °C; ^1^H NMR (600 MHz, DMSO-*d_6_*) *δ* 8.24 (d, *J* = 3.0 Hz, 1H, H_2_-pyridine), 7.83 (dd, *J* = 9.0, 3.0 Hz, 1H, H_4_-pyridine), 7.27 (d, *J* = 7.8 Hz, 1H, NHCO), 6.89 (d, *J* = 9.0 Hz, 1H, H_5_-pyridine), 4.89–4.85 (m, 1H, H_5_-oxazolidone), 4.28–4.16 (m, 2H, OCH_2_), 4.11–4.08 (m, 1H, H_4_-oxazolidone), 3.86–3.72 (m, 1H, H_4_-oxazolidone), 3.71–3.69 (m, 4H, morpholine), 3.40–3.39 (m, 4H, morpholine), 1.75–1.59 (m, 4H, cyclohexane), 1.55–1.49 (m, 2H, cyclohexane), 1.29–1.20 (m, 3H, cyclohexane), 1.18–1.06 (m, 1H, cyclohexane), 1.04–0.96 (m, 1H, cyclohexane); ^13^C NMR (150 MHz, DMSO-*d_6_*) *δ* 159.3, 157.4, 154.6, 146.4, 135.2, 114.8, 106.7, 79.7, 65.8, 47.6, 44.8, 42.4, 36.9, 33.2, 25.3, 24.4; ES-MS: *m/z* calcd. for C_20_H_28_N_4_O_5_ [M + H]^+^: 405.2; found: 405.174.

*(R)-(3-(6-morpholinopyridin-3-yl)-2-oxooxazolidin-5-yl)methyl (4-chlorophenyl)carbamate* (**11g**).

A red solid; yield 58%; m. p. 185.0–187.9 °C; ^1^H NMR (600 MHz, DMSO-*d*_6_) *δ* 8.90 (s, 1H, NHCO), 8.25 (d, *J* = 3.0 Hz, 1H, H_2_-pyridine), 7.84 (dd, *J* = 9.0, 3.0 Hz, 1H, H_4_-pyridine), 7.50–7.46 (m, 2H, benzene), 7.35–7.31 (m, 2H, benzene), 6.89 (d, *J* = 9.0 Hz, 1H, H_5_-pyridine), 4.99–4.96 (m, 1H, H_5_-oxazolidone), 4.12–4.35 (m, 2H, OCH_2_), 4.15–4.12 (m, 1H, H_4_-oxazolidone), 3.85–3.82 (m, 1H, H_4_-oxazolidone), 3.71–3.69 (m, 4H, morpholine), 3.43–3.38 (m, 4H, morpholine); ^13^C NMR (150 MHz, DMSO-*d*_6_) *δ* 156.7, 154.9, 153.5, 152.8, 139.1, 129.9, 129.1, 126.8, 126.0, 120.3, 107.5, 71.5, 66.4, 65.1, 46.8, 46.0; ES-MS: *m/z* calcd. for C_20_H_21_ClN_4_O_5_ [M + H]^+^: 433.1; found: 433.098.

*(R)-(3-(6-morpholinpyridin-3-yl)-2-oxooxazolidin-5-yl)methyl methanesulfonate* (**11h**).

A yellow solid; yield 75%; m. p. 184.2—185.9 °C; ^1^H NMR (600 MHz, DMSO-*d*_6_), *δ*: 8.23 (d, *J* = 3.0 Hz, 1H, H_2_-pyridine), 7.82 (dd, *J* = 9.0, 3.0 Hz, 1H, H_4_-pyridine), 6.90 (d, *J* = 9.0 Hz, 1H, H_5_-pyridine), 5.02–4.96 (m, 1H, H_5_-oxazolidone), 4.53–4.44 (m, 2H, OCH_2_), 4.17–4.12 (m, 1H, H_4_-oxazolidone), 3.80–3.76 (m, 1H, H_4_-oxazolidone), 3.71– 3.68 (m, 4H, morpholine), 3.41– 3.39 (m, 4H, morpholine), 3.26 (s, 3H, CH_3_); ^13^C NMR (150 MHz, DMSO-*d*_6_) *δ* 156.8, 154.7, 139.2, 130.1, 126.6, 107.5, 70.8, 70.3, 66.4, 46.6, 45.9, 37.3; ES-MS: *m/z* calcd. for C_14_H_19_N_3_O_6_S [M + H]^+^: 358.1; found: 358.096.

*(R)-(3-(6-morpholinpyridin-3-yl)-2-oxooxazolidin-5-yl)methyl benzenesulfonate* (**11i**).

Red oil; yield 55%; ^1^H NMR (600 MHz, DMSO-*d*_6_) *δ* 8.13 (d, *J* = 3.0 Hz, 1H, H_2_-pyridine), 7.94 (dd, *J* = 9.0, 3.0 Hz, 1H, H_4_-pyridine), 7.85–7.80 (m, 1H, benzene), 7.63–7.59 (m, 2H, benzene), 7.36–7.26 (m, 2H, benzene), 7.16 (d, *J* = 9.0 Hz, 1H, H_5_-pyridine), 5.00–4.95 (m, 1H, H_5_-oxazolidone), 4.41–4.33 (m, 1H, OCH_2_), 4.19–4.11 (m, 1H, OCH_2_), 3.83–3.79 (m, 2H, H_4_-oxazolidone), 3.74–3.72 (m, 4H, morpholine), 3.51–3.48 (m, 4H, morpholine); ^13^C NMR (150 MHz, DMSO-*d*_6_) *δ* 159.9, 154.9, 146.9, 141.0, 135.7, 132.9, 129.7, 126.9, 115.1, 107.2, 79.1, 66.3, 46.1 45.2, 37.6; ES-MS: *m/z* calcd. for C_19_H_21_N_3_O_6_S [M + H]^+^: 420.2; found: 420.111.

*(R)-(3-(6-(4-(cyclohexanecarbonyl)piperazin-1-yl)pyridin-3-yl)-2-oxooxazolidin-5-yl)methyl methanesulfonate* (**12a**).

A white solid; yield 73%; m. p. 111.3–112.5 °C; ^1^H NMR (600 MHz, DMSO-*d*_6_) δ 8.23 (d, *J* = 3.0 Hz, 1H, H_2_-pyridine), 7.84 (dd, *J* = 9.0, 3.0 Hz, 1H, H_4_-pyridine), 6.94 (d, *J* = 9.0 Hz, 1H, H_5_-pyridine), 5.02–4.98 (m, 1H, H_5_-oxazolidone), 4.54–4.44 (m, 2H, OCH_2_), 4.15–4.13 (m, 1H, H_4_-oxazolidone), 3.82–3.73 (m, 1H, H_4_-oxazolidone), 3.61–3.59 (m, 4H, piperazine), 3.50–3.48 (m, 4H, piperazine), 3.26 (s, 3H, CH_3_), 1.77–1.65 (m, 11H, cyclohexane); ^13^C NMR (150 MHz, DMSO-*d*_6_) *δ* 174.1, 154.7, 139.2, 130.3, 128.9, 126.5, 107.9, 70.8, 70.3, 46.6, 46.0, 45.4, 44.8, 37.3, 29.6, 26.1, 25.6; ES-MS: *m/z* calcd. for C_21_H_30_N_4_O_6_S [M + H]^+^: 467.5; found: 467.183.

*(R)-(2-oxo-3-(6-(4-(4-(trifluoromethyl)benzoyl)piperazin-1-yl)pyridin-3-yl)oxazolidin-5-yl)methyl methanesulfonate* (**12b**).

A white solid; yield 72%; m. p. 164.2–165.3 °C; ^1^H NMR (600 MHz, DMSO-*d*_6_) *δ* 8.24 (d, *J* = 3.0 Hz, 1H, H_2_-pyridine), 7.87–7.82 (m, 3H, H_4_-pyridine and benzene), 7.68 (d, *J* = 7.8 Hz, 2H, benzene), 6.94 (d, *J* = 9.0 Hz, 1H, H_5_-pyridine), 5.02–4.98 (m, 1H, H_5_-oxazolidone), 4.54–4.44 (m, 2H, OCH_2_), 4.17–4.13 (m, 1H, H_4_-oxazolidone), 3.80–3.60 (m, 5H, H_4_-oxazolidone and piperazine), 3.49–3.36 (m, 4H, piperazine), 3.26 (s, 3H, CH_3_); ^13^C NMR (150 MHz, DMSO-*d*_6_) *δ* 168.3, 166.4, 156.3 (d, *J* = 231.1 Hz), 154.8, 140.5, 139.3, 130.2, 128.3, 126.6, 126.0, 107.8, 70.8, 70.3, 46.6, 41.7, 40.5, 37.3.; ES-MS: *m/z* calcd. for C_22_H_23_F_3_N_4_O_6_S [M + H]^+^: 529.5; found: 529.104.

*(R)-(2-oxo-3-(6-(4-(3-phenylpropanoyl)piperazin-1-yl)pyridin-3-yl)oxazolidin-5-yl)methyl methanesulfonate* (**12c**).

A yellow solid; yield 74%; m. p. 133.0–135.0 °C; ^1^H NMR (600 MHz, DMSO-*d*_6_) *δ* 8.22 (d, *J* = 3.0 Hz, 1H, H_2_-pyridine), 7.82 (dd, *J* = 9.0, 3.0 Hz, 1H, H_4_-pyridine), 7.31–7.24 (m, 5H, benzene), 6.91 (d, *J* = 9.0 Hz, 1H, H_5_-pyridine), 5.014.97 (m, 1H, H_5_-oxazolidone), 4.54–4.44 (m, 2H, OCH_2_), 4.16–4.13 (m, 1H, H_4_-oxazolidone), 3.79–3.77 (m, 1H, H_4_-oxazolidone), 3.58–3.51 (m, 4H, piperazine), 3.43–3.40 (m, 4H, piperazine), 3.26 (s, 3H, CH_3_), 2.86–2.76 (m, 2H, CH_2_), 2.70–2.62 (m, 2H, CH_2_).; ^13^C NMR (150 MHz, DMSO-*d*_6_) *δ* 170.6, 156.3, 154.7, 141.9, 139.4, 130.2, 128.9, 128.7, 126.4, 126.3, 107.7, 70.8, 46.6, 45.6, 44.9, 41.1, 37.3, 34.5, 31.2; ES-MS: *m/z* calcd. for C_23_H_28_N_4_O_6_S [M + H]^+^: 489.56; found: 489.163.

*(R,E)-(2-oxo-3-(6-(4-(3-(pyridin-3-yl)acryloyl)piperazin-1-yl)pyridin-3-yl)oxazolidin-5-yl)methyl methanesulfonate* (**12d**).

A yellow solid; yield 75%; m. p. 104.5–106.7 °C; ^1^H NMR (600 MHz, DMSO-*d*_6_) *δ* 8.91 (d, *J* = 2.4 Hz, 1H, pyridine), 8.56 (dd, *J* = 4.8, 1.8 Hz, 1H, pyridine), 8.25 (d, *J* = 3.0 Hz, 1H, H_2_-pyridine (B ring)), 8.23–8.20 (m, 1H, pyridine), 7.88–7.83 (m, 1H, H_4_-pyridine (B ring)), 7.58–7.46 (m, 3H, pyridine and alkene), 6.98–6.95 (m, 1H, H_5_-pyridine (B ring)), 5.02–4.79 (m, 1H, H_5_-oxazolidone), 4.54–4.46 (m, 1H, OCH_2_), 4.19–4.05 (m, 1H, OCH_2_), 3.88–3.66 (m, 6H, H_4_-oxazolidone and piperazine), 3.56–3.51 (m, 4H, piperazine), 3.27 (s, 3H, CH_3_). ^13^C NMR (150 MHz, DMSO-*d*_6_) *δ* 164.7, 156.1, 155.1, 150.6, 150.0, 138.8, 135.1, 131.4, 130.2, 130.0, 126.9, 124.3, 120.8, 107.8, 73.0, 72.2, 70.8, 59.2, 46.9, 37.3; ES-MS: *m/z* calcd. for C_22_H_25_N_5_O_6_S [M + H]^+^: 488.5; found: 489.181.

*(R)-(3-(6-(4-(cyclohexylcarbamoyl)piperazin-1-yl)pyridin-3-yl)-2-oxooxazolidin-5-yl)methyl methanesulfonate* (**12e**).

A white solid; yield 61%; m. p. 195.0–197.6 °C; ^1^H NMR (600 MHz, DMSO-*d*_6_) *δ* 8.22 (d, *J* = 3.0 Hz, 1H, H_2_-pyridine), 7.82 (dd, *J* = 9.0, 3.0 Hz, 1H, H_4_-pyridine), 6.93 (d, *J* = 9.0 Hz, 1H, H_5_-pyridine), 6.25 (d, *J* = 7.8 Hz, 1H, NHCON), 5.02–4.97 (m, 1H, H_5_-oxazolidone), 4.54–4.43 (m, 2H, OCH_2_), 4.18–4.13 (m, 1H, H_4_-oxazolidone), 3.80–3.77 (m, 1H, H_4_-oxazolidone), 3.46–3.37 (m, 8H, piperazine), 3.27 (s, 3H, CH_3_), 1.78–1.73 (m, 2H, cyclohexane), 1.71–1.67 (m, 2H, cyclohexane), 1.60–1.55 (m, 1H, cyclohexane), 1.29–1.13 (m, 5H, cyclohexane), 1.12–1.03 (m, 1H, cyclohexane); ^13^C NMR (150 MHz, DMSO-*d*_6_) *δ* 157.3, 156.6, 154.7, 139.4, 130.1, 126.3, 107.7, 70.8, 70.3, 49.6, 46.6, 45.4, 43.5, 37.3, 33.6, 25.9, 25.6; ES-MS: *m/z* calcd. for C_21_H_31_N_5_O_6_S [M + H]^+^: 482.2; found: 482.203.

*(R)-((3-(6-(4-(4-chlorophenyl)carbamoyl)piperazin-1-yl)pyridin-3-yl)-2-oxooxazolidin-5-yl)methyl methanesulfonate* (**12f**).

A white solid; yield 53%; m. p. 210.5—212.9 °C; ^1^H NMR (600 MHz, DMSO-*d*_6_) *δ* 8.73 (s, 1H, NHCON), 8.24 (d, *J* = 3.0 Hz, 1H, H_2_-pyridine), 7.84 (dd, *J* = 9.0, 3.0 Hz, 1H, H_4_-pyridine), 7.55–7.50 (m, 2H, benzene), 7.31–7.27 (m, 2H, benzene), 6.97 (d, *J* = 9.0 Hz, 1H, H_5_-pyridine), 5.03–4.98 (m, 1H, H_5_-oxazolidone), 4.53–4.46 (m, 2H, OCH_2_), 4.17–4.14 (m, 1H, H_4_-oxazolidone), 3.81–3.78 (m, 1H, H_4_-oxazolidone), 3.58–3.56 (m, 4H, piperazine), 3.53–3.51 (m, 4H, piperazine), 3.27 (s, 3H, CH_3_); ^13^C NMR (150 MHz, DMSO-*d*_6_) *δ* 156.5, 155.3, 154.7, 140.0, 139.4, 130.2, 128.7, 126.4, 125.8, 121.5, 107.8, 70.8, 70.3, 46.6, 45.4, 43.8, 37.3; ES-MS: *m/z* calcd. for C_21_H_24_ClN_5_O_6_S [M + H]^+^:510.1; found: 510.122.

*(R)-(3-(6-(4-((4-fluorophenyl)sulfonyl)piperazin-1-yl)pyridin-3-yl)-2-oxooxazolidin-5-yl)methyl methanesulfonate* (**12g**).

A white solid; yield 58%; m. p. 215.7—218.3 °C; ^1^H NMR (600 MHz, DMSO-*d*_6_) *δ* 8.19 (d, *J* = 3.0 Hz, 1H, H_2_-pyridine), 7.87–7.82 (m, 2H, benzene), 7.81 (dd, *J* = 9.0, 3.0 Hz, 1H, H_4_-pyridine), 7.53–7.46 (m, 2H, benzene), 6.90 (d, *J* = 9.0 Hz, 1H, H_5_-pyridine), 5.00–4.96 (m, 1H, H_5_-oxazolidone), 4.52–4.42 (m, 2H, OCH_2_), 4.14–4.11 (m, 1H, H_4_-oxazolidone), 3.77–3.75 (m, 1H, H_4_-oxazolidone), 3.60–3.55 (m, 4H, piperazine), 3.26 (s, 3H, CH_3_), 3.00–2.98 (m, 4H, piperazine); ^13^C NMR (150 MHz, DMSO-*d*_6_) *δ* 165.2 (d, *J* = 252.2 Hz), 155.7, 154.7, 138.9, 131.2, 130.2, 126.8, 117.3, 108.1, 70.8, 70.3, 46.5, 45.9, 44.9, 37.3; ES-MS: *m/z* calcd. for C_20_H_23_FN_4_O_7_S_2_ [M + H]^+^: 515.1; found: 515.107.

*(R)-(3-(6-(4-((2,4-difluorophenyl)sulfonyl)piperazin-1-yl)pyridin-3-yl)-2-oxooxazolidin-5-yl)methyl methanesulfonat**e*** (**12h**).

A white solid; yield 71%; m. p. 214.6—215.6 °C; ^1^H NMR (600 MHz, DMSO-*d*_6_) *δ* 8.20 (d, *J* = 3.0 Hz, 1H, H_2_-pyridine), 7.82 (dd, *J* = 9.0, 3.0 Hz, 1H, H_4_-pyridine), 7.72–7.67 (m, 1H, benzene), 7.11 (dd, *J* = 12.6, 2.4 Hz, 1H, benzene), 6.98 (dd, *J* = 9.0, 2.4 Hz, 1H, benzene), 6.91 (d, *J* = 9.0 Hz, 1H, H_5_-pyridine), 5.01–4.96 (m, 1H, H_5_-oxazolidone), 4.52–4.41 (m, 2H, OCH_2_), 4.15–4.11 (m, 1H, H_4_-oxazolidone), 3.82–3.68 (m, 1H, H_4_-oxazolidone), 3.58–3.56 (m, 4H, piperazine), 3.26 (s, 3H, CH_3_), 3.10–3.07 (m, 4H, piperazine). ^13^C NMR (150 MHz, DMSO-*d*_6_) *δ* 165.3, 160.2 (d, *J_C-F_* = 253.1 Hz), 155.9, 154.7 (d, *J* = 176.7 Hz), 139.1, 132.8, 130.1, 126.7, 115.4, 111.5, 108.1, 103.8, 70.8, 70.3, 56.8, 46.5, 45.6, 45.1; ES-MS: *m/z* calcd. for C_20_H_22_F_2_N_4_O_7_S_2_ [M + H]^+^: 533.5; found: 533.206.

Raw data for the above products are presented in Supplementary Materials (Figure S17-S52).

### 3.3. Antibacterial Activity Assay

#### 3.3.1. Minimum Inhibitory Concentration (MIC) Study

According to the standard procedure recommended by the Clinical Laboratory Standards Institute (CLSI) [37], the minimum inhibitory concentration (MIC) in vitro of all synthesized compounds for Gram-positive strains was evaluated on 96-well microplates by the broth microdilution technique to screen for antibacterial activities. In Mueller–Hinton (MH) sterile medium, a series of compound solutions was prepared by double-fold dilution method from 0.5 to 256 μg/mL. After that, 100 μL diluted standard inoculum (1 × 10^6^ CFU/mL) was added into the gradient solution. The 96-well plates were cultured at 37 °C for 16–18 h, and the MIC of each well was considered to be the lowest concentration that could significantly inhibit the growth of bacteria. The untreated inoculum was used as a negative control and linezolid was used as a positive control [38].

#### 3.3.2. Bacterial Inhibitory Zone Test

The bacterial inhibitory zone of **12e** against *S. aureus* (ATCC 25923) was determined by pore diffusion method [39]. A total of 100 µL of fresh culture was dispersed on sterile petri dishes containing nutrient AGAR media. The holes were made in the petri plates using 100 µL micro pipette tips and injected with varied concentrations of **12e** (160, 320, 640, and 1600 µg/mL) and 40 µg/mL of control (linezolid), following which the petri plates were kept overnight at 37 °C. After incubation, a band was formed around the holes by inhibiting biological growth.

#### 3.3.3. Growth Kinetic Study

Based on the previous result of antibacterial activity, compound **12e** was selected for further research. According to the literature [36], the growth kinetics of Bacillus subtilis (BNCC 109047) was determined after treatment with compound **12e** and linezolid. The log-phase strain was diluted to 10^6^–10^7^ CFU/mL in LB broth containing compound **12e** at a series of concentrations (0, 1/4, 1/2, 1 and 2 × MIC), then incubated at 37 °C and 150 rpm. Linezolid at the concentration of 2 μg/mL was used as a positive control. At the beginning, culture mixture (3 mL) was removed from each sample solution at intervals of 1 h, and turbidity was measured by ultraviolet spectrophotometer at 600 nm. When the O.D. value became stable, the time interval was gradually increased and the time–kill curve was drawn.

### 3.4. Binding Mode Study

Molecular docking assay was performed using the AutoDock 4.2.6^®^ software. The X-ray crystal 3D structure of the 50S ribosomal subunit (PDB ID: 3CPW) was obtained from the Protein Data Bank (PDB) database. The ligands and proteins were removed from the structure and RNA chains were retained using PyMOL 1.5.0.3. The modified structure was prepared for further molecular docking. 2D structures of the ligands were built with the aid of ChemDraw 19.0 and the 3D structures were established by Chem3D 19.0 software. The docking results were analyzed and visualized with PyMOL 1.5.0.3.

### 3.5. Anthelmintic Activity Assay

Due to the high similarity between adult Indian earthworms (*P. posthuma*) and intestinal roundworms (*A. bricoides*) in terms of anatomical and physiological characteristics [40], anthelmintic activities of all compounds were evaluated [41,42].

Before use, the earthworms with an average length of 6–10 cm and width of 0.2–0.3 cm were cleaned with normal saline solution [41], and the target compounds and positive control drug (albendazole) were dissolved with the minimum amount of DMSO and diluted with normal saline solution.

Then, the tested solution with a massive concentration of 2 g/L was prepared and placed in a Petri dish (d = 6 cm). After being divided into different groups with six (6) worms, they were transferred to Petri dishes with sterile forceps and the paralysis and death time were monitored and recorded with stopwatches for 1 h. Earthworms tested in standard saline (0.9% *w/v*) were taken as the control group. The paralysis time was considered as the time when the worm showed no obvious movement at rest or under mild vibration. In a similar way, death time was considered when there was no obvious movement after severe shaking and exposure to 50 °C water [41,42]. The mean paralysis and death times were was used to reflect the anthelmintic effect of these compounds; the shorter time, the better the effect.

## 4. Conclusions

Seventeen novel 3-(3-pyridyl)-oxazolidone-5-methyl derivatives were designed and synthesized, and their structures were identified by ^1^H NMR, ^13^C NMR and MS. The primary screening of antibacterial activity, which was tested on *Staphylococcus aureus* (ATCC25923), *Streptococcus pneumoniae* (ATCC49619), *Bacillus subtilis* (BNCC109047) and *Staphylococcus epidermidis* (BNCC186652), showed that most of the compounds had certain antibacterial activity, among which compound **12a** and **12e** had the best antimicrobial activity. In a further bactericidal kinetic assay, **12e** was mainly interfering in the logarithmic phase of bacteria in a concentration-dependent manner. Molecular docking between the **12e** and the 50S ribosomal subunit was studied to predict the mechanism of action. In addition, the anthelmintic activity test demonstrated that the compound **11b** had the most significant anthelmintic effect. These results provide a preliminary basis for the development of novel pyridine heterocyclic compounds as antibacterial and anthelmintic agents. Further structural optimization and biological activity confirmation of the action model are in progress.

## Figures and Tables

**Table 1:** In vitro antibacterial activity of target derivatives (MIC: μg/mL).

Compound	Antimicrobial Screening MIC (μg/mL)			
	*S. Aureus* Atcc 25923	*S. Pneumoniae* Atcc 49619	*B. Subtilis* Bncc 109047	*S. Epidermidis* Bncc 186652
**11a**	128	>256	256	>256
**11b**	128	>256	128	>256
**11c**	>256	>256	128	>256
**11d**	>256	>256	256	>256
**11e**	>256	>256	>256	>256
**11f**	256	128	128	128
**11g**	>256	256	>256	128
**11h**	128	128	64	128
**11i**	>256	256	>256	>256
**12a**	32	32	32	16
**12b**	128	128	128	128
**12c**	64	32	32	32
**12d**	32	64	64	64
**12e**	32	32	16	32
**12f**	128	128	64	64
**12g**	128	32	32	128
**12h**	128	128	128	128
**Linezolid**	2	2	2	2

MIC: minimal inhibit concentration.

**Table 2:** Anthelmintic Activities of Target Compounds.

Compound	Mean Paralysis Time/Min	Mean Death Time/Min
**11a**	11.0 ± 1.8	35.9 ± 5.2
**11b**	3.6 ± 0.2	8.1 ± 0.8
**11c**	28.3 ± 5.8	>60
**11d**	8.6 ± 1.4	35.6 ± 6.0
**11e**	13.0 ± 0.4	>60
**11f**	11.6 ± 1.0	>60
**11g**	13.7 ± 1.7	52.1 ± 7.0
**11h**	14.8 ± 0.9	>60
**11i**	18.8 ± 2.7	>60
**12a**	17.4 ± 1.7	55.2 ± 2.9
**12b**	22.6 ± 4.3	>60
**12c**	19.5 ± 2.2	50.3 ± 1.9
**12d**	18.3 ± 2.1	55.2 ± 5.0
**12e**	33.1 ± 2.3	>60
**12f**	35.8 ± 2.3	>60
**12g**	32.6 ± 3.6	>60
**12h**	15.6 ± 1.7	>60
Albendazole	15.0 ± 1.5	40.8 ± 4.3

## Data Availability

Not applicable.

## Supplementary Materials

Figure S1: Structure and Numbering of **11a**, Figure S2: ^1^H NMR Spectrum of **11a**, Figure S3: ^13^C NMR Spectrum of **11a**. Figure S4: ES-MS for compound **11a**, Figure S5: ^1^H NMR Spectrum of **11b**, Figure S6: ^13^C NMR Spectrum of **11b**. Figure S7: ES-MS for compound **11b**, Figure S8: ^1^H NMR Spectrum of **11c**, Figure S9: ^13^C NMR Spectrum of **11c**. Figure S10: ES-MS for compound **11c**, Figure S11: ^1^H NMR Spectrum of **11d**, Figure S12: ^13^C NMR Spectrum of **11d**. Figure S13: ES-MS for compound **11d**, Figure S14: ^1^H NMR Spectrum of **11e**, Figure S15: ^13^C NMR Spectrum of **11e**. Figure S16: ES-MS for compound **11e**, Figure S17: ^1^H NMR Spectrum of **11f**, Figure S18: ^13^C NMR Spectrum of **11f**. Figure S19: ES-MS for compound **11f**, Figure S20: ^1^H NMR Spectrum of **11g**, Figure S21: ^13^C NMR Spectrum of **11g**. Figure S22: ES-MS for compound **11g**, Figure S23: ^1^H NMR Spectrum of **11h**, Figure S24: ^13^C NMR Spectrum of **11h**. Figure S25: ES-MS for compound **11h**, Figure S26: ^1^H NMR Spectrum of **11i**, Figure S27: ^13^C NMR Spectrum of **11i**. Figure S28: ES-MS for compound **11i**, Figure S29: ^1^H NMR Spectrum of **12a**, Figure S30: ^13^C NMR Spectrum of **12a**. Figure S31: ES-MS for compound **12a**, Figure S32: ^1^H NMR Spectrum of **12b**, Figure S33: ^13^C NMR Spectrum of **12b**. Figure S34: ES-MS for compound **12b**, Figure S35: ^1^H NMR Spectrum of **12c**, Figure S36: ^13^C NMR Spectrum of **12c**. Figure S37: ES-MS for compound **12c**, Figure S38: ^1^H NMR Spectrum of **12d**, Figure S39: ^13^C NMR Spectrum of **12d**. Figure S40: ES-MS for compound **12d**, Figure S41: ^1^H NMR Spectrum of **12e**, Figure S42: ^13^C NMR Spectrum of **12e**. Figure S43: ES-MS for compound **12e**, Figure S44: ^1^H NMR Spectrum of **12f**, Figure S45: ^13^C NMR Spectrum of **12f**. Figure S46: ES-MS for compound **12f**, Figure S47: ^1^H NMR Spectrum of **12g**, Figure S48: ^13^C NMR Spectrum of **12g**. Figure S49: ES-MS for compound **12g**, Figure S50: ^1^H NMR Spectrum of **12h**, Figure S51: ^13^C NMR Spectrum of **12h**. Figure S52: ES-MS for compound **12h.**

## Sample Availability

Samples of the compounds are not available from the authors.
