# Supplementary material for: Synthesis, Antibacterial and Anthelmintic Activity of Novel 3-(3-Pyridyl)-oxazolidinone-5-methyl Ester Derivatives
Source: Molecules. 2022 Feb 7;27(3):1103. doi: 10.3390/molecules27031103 (PMC8839302; doi:10.3390/molecules27031103)
Supplement: Supplementary file 1 [file molecules-27-01103-s001.zip › molecules-1549096-supplementary.pdf]

Supplementary Materials Design, Synthesis and Evaluation Biological Activity of

3-(3-pyridyl)-oxazolidinone-5-methyl Ester Deriviatives

JIN Bo[1], CHEN Jia-yi[1], SHENG Zun-lai[1,2], SUN Meng-qing[1,2] and YANG Hong-liang[1,2*]

1. College of Veterinary Medicine, Northeast Agricultural University, Harbin 150030, Heilongjiang, China;

2. Heilongjiang Key Laboratory for Animal Disease Control and Pharmaceutical Development, Harbin 150030, Heilongjiang, China

*Email: hongl_yang@126.com

Structure and Numbering of Target Compound

Take compound **11a** for an example, the numbering of final product is given and shown in Figure S1.

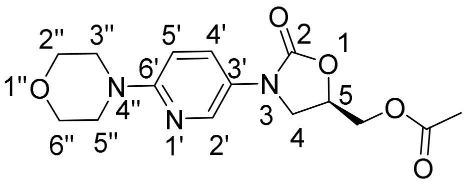

11a

Figure S1. Structure and Numbering of **11a**

^{1}H NMR, ^{13}C NMR and ES-MS spectra of 11a-i and 12a-h

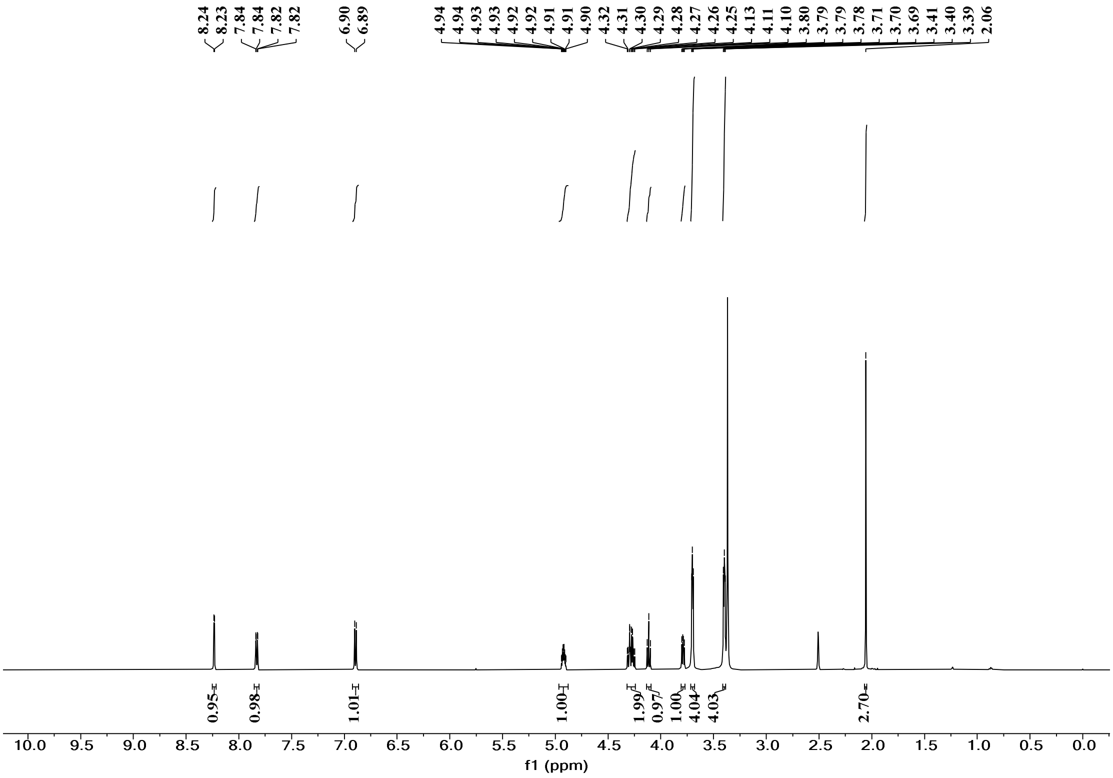

Figure S2. ^{1}H NMR Spectrum (DMSO-d_6, 600 MHz) of **11a**.

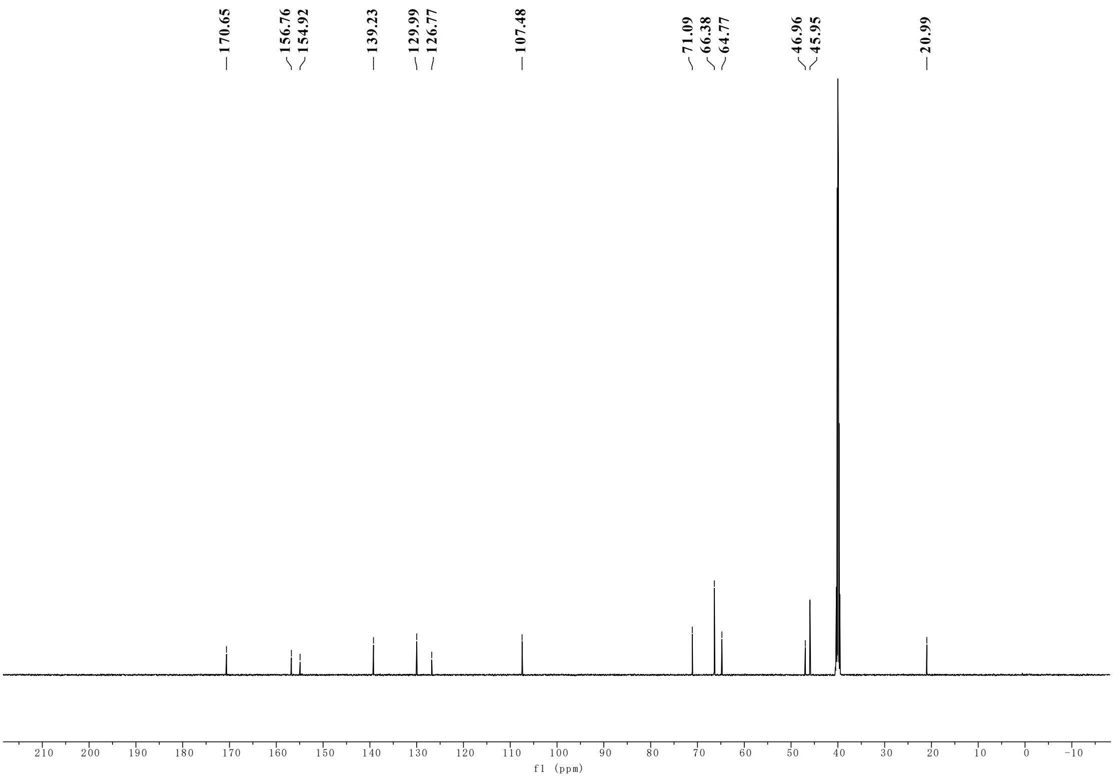

Figure S3. ^{13}C NMR Spectrum (DMSO-d_6, 151 MHz) of **11a**.

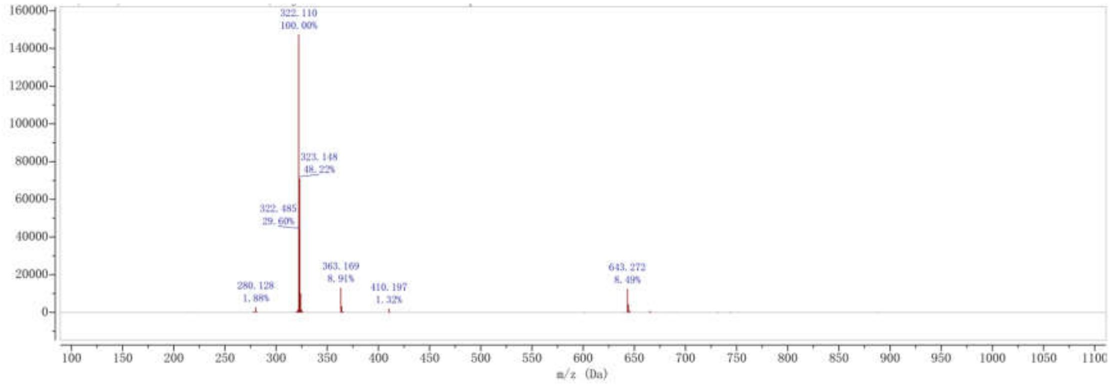

Figure S4. ES-MS for compound **11a**.

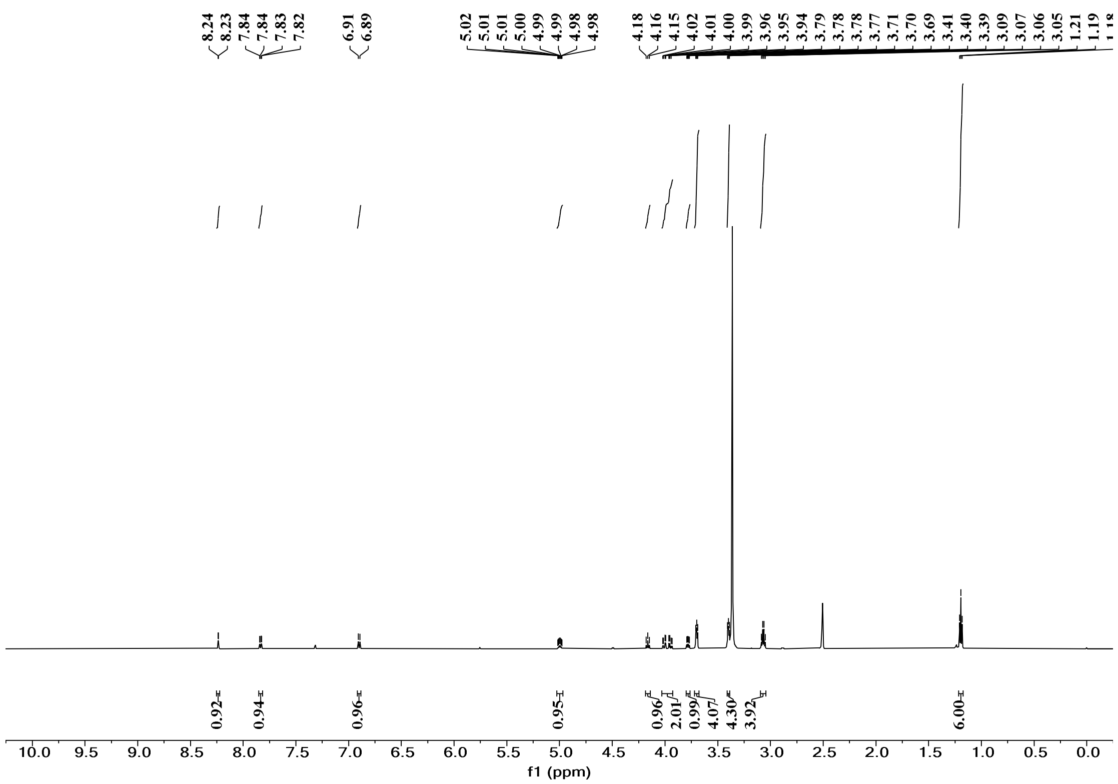

Figure S5. ^{1}H NMR Spectrum (DMSO-d_6, 600 MHz) of **11b**.

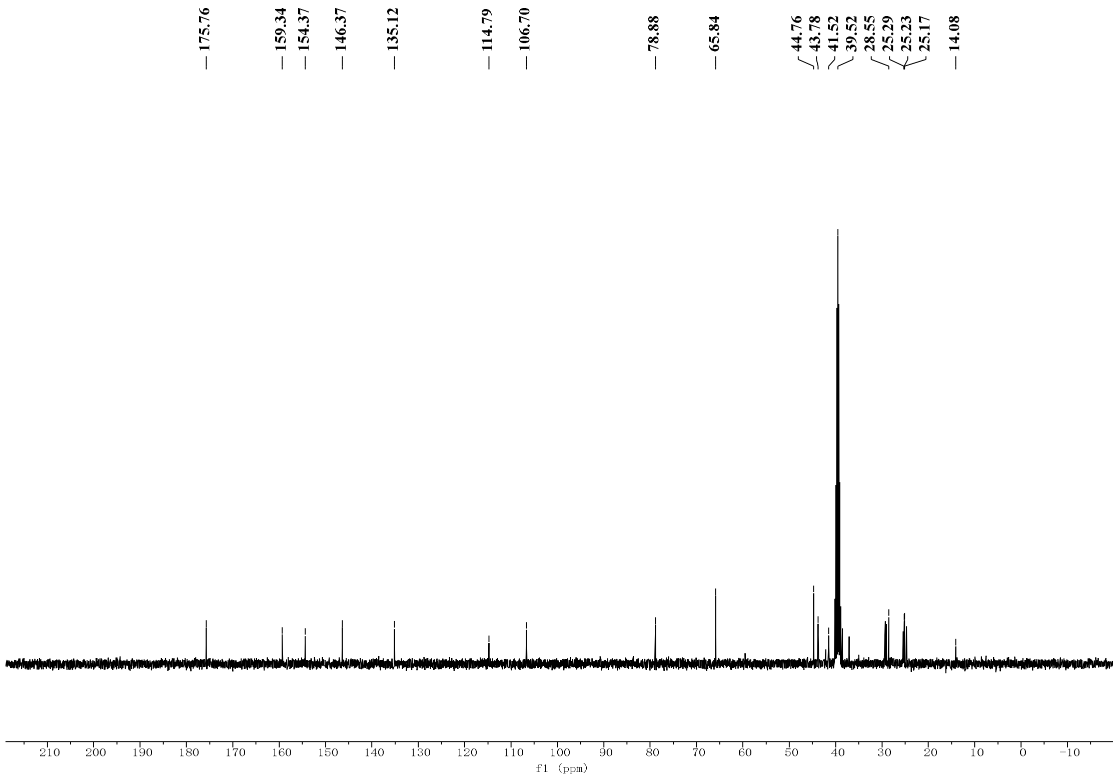

Figure S6. ^{13}C NMR Spectrum (DMSO-d_6, 151 MHz) of **11b**.

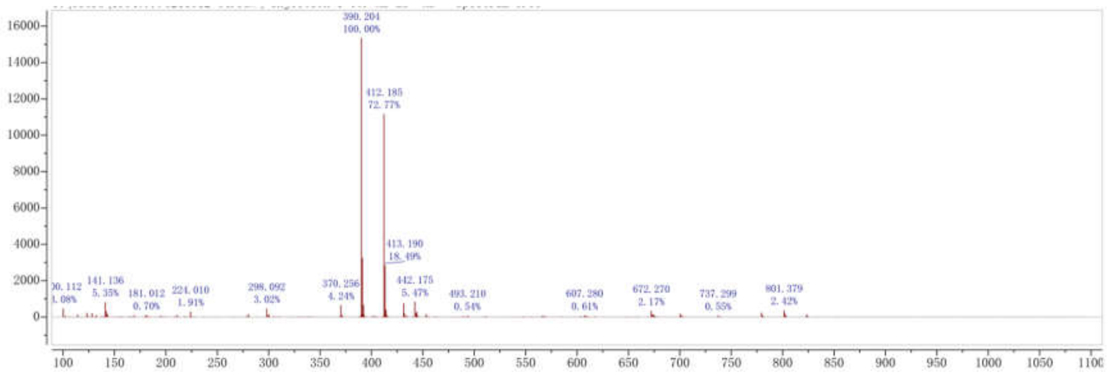

Figure S7. ES-MS for compound **11b**.

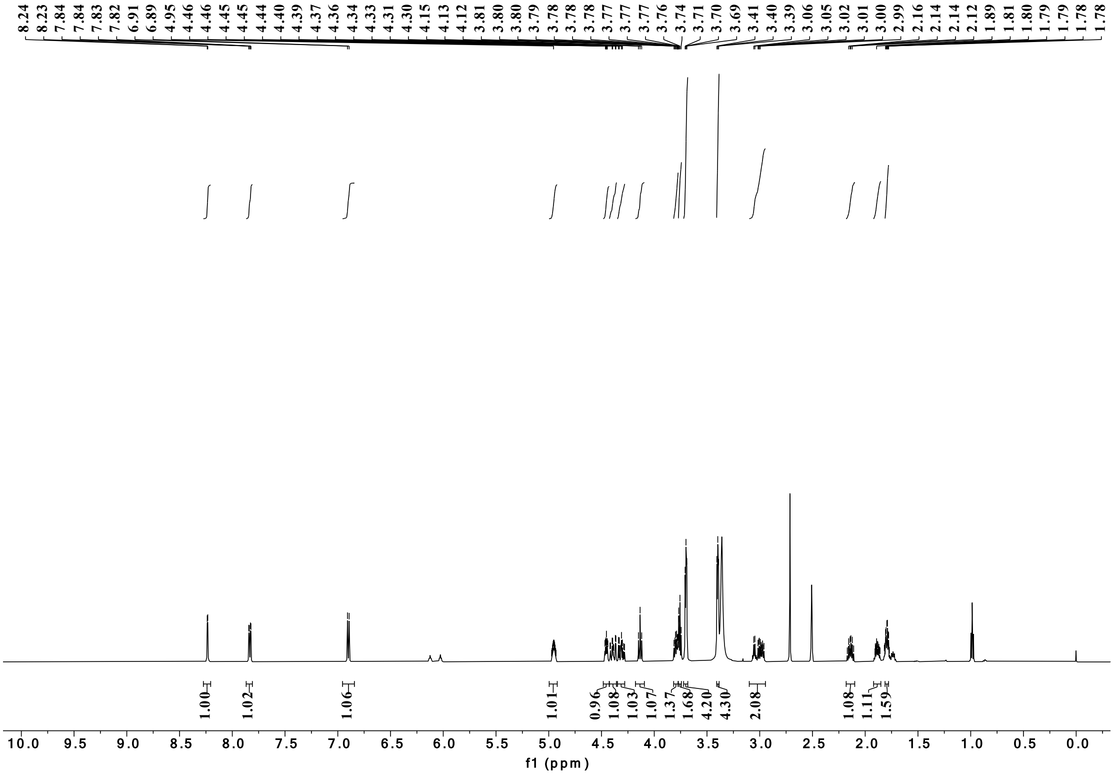

Figure S8. ^{1}H NMR Spectrum (DMSO-d_6, 600 MHz) of **11c**.

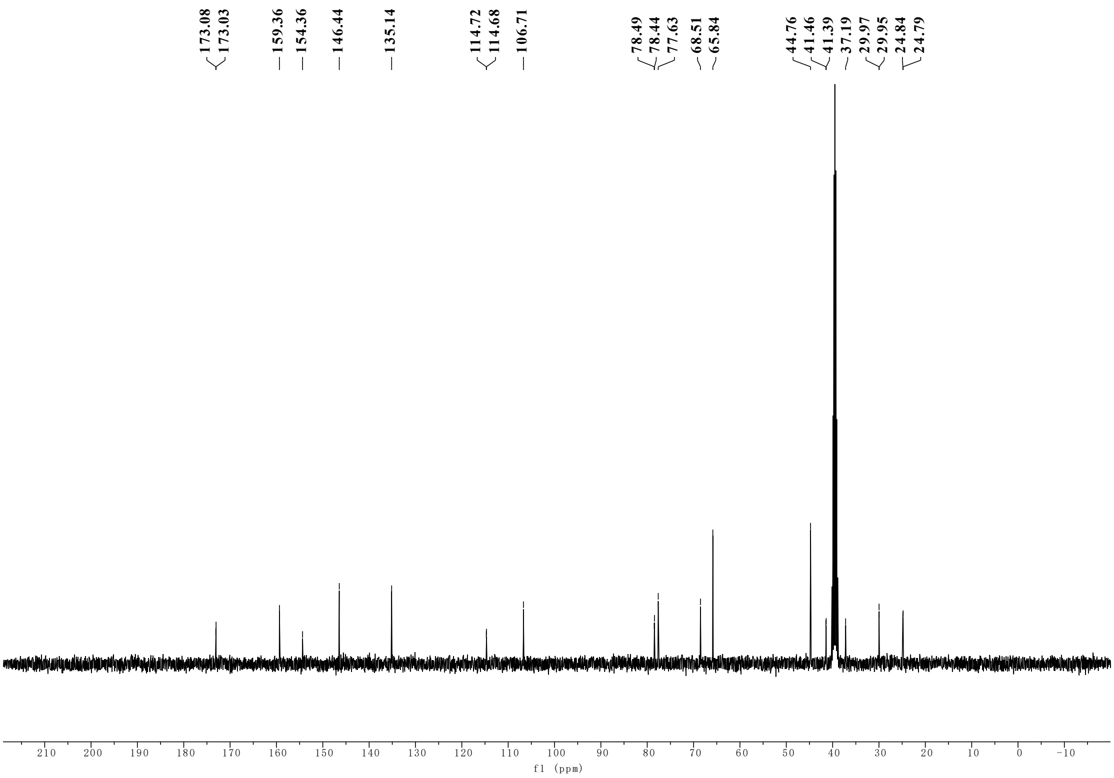

Figure S9. ^{13}C NMR Spectrum (DMSO-d_6, 151 MHz) of **11c**.

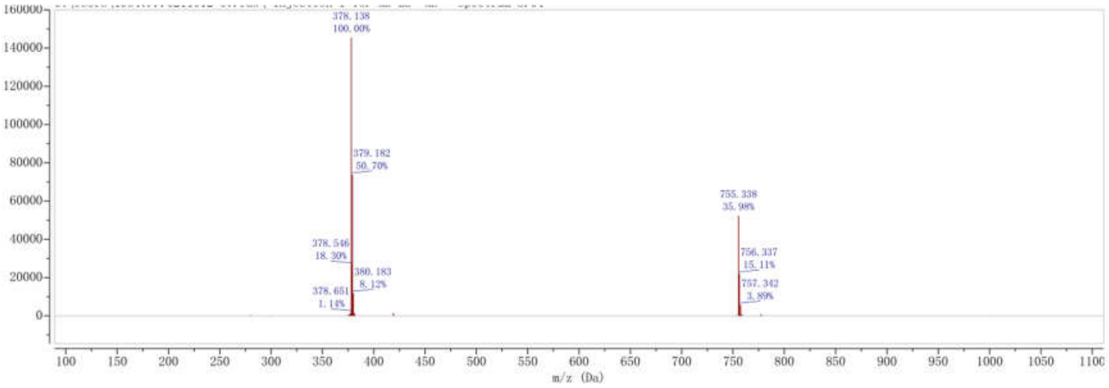

Figure S10. ES-MS for compound **11c**.

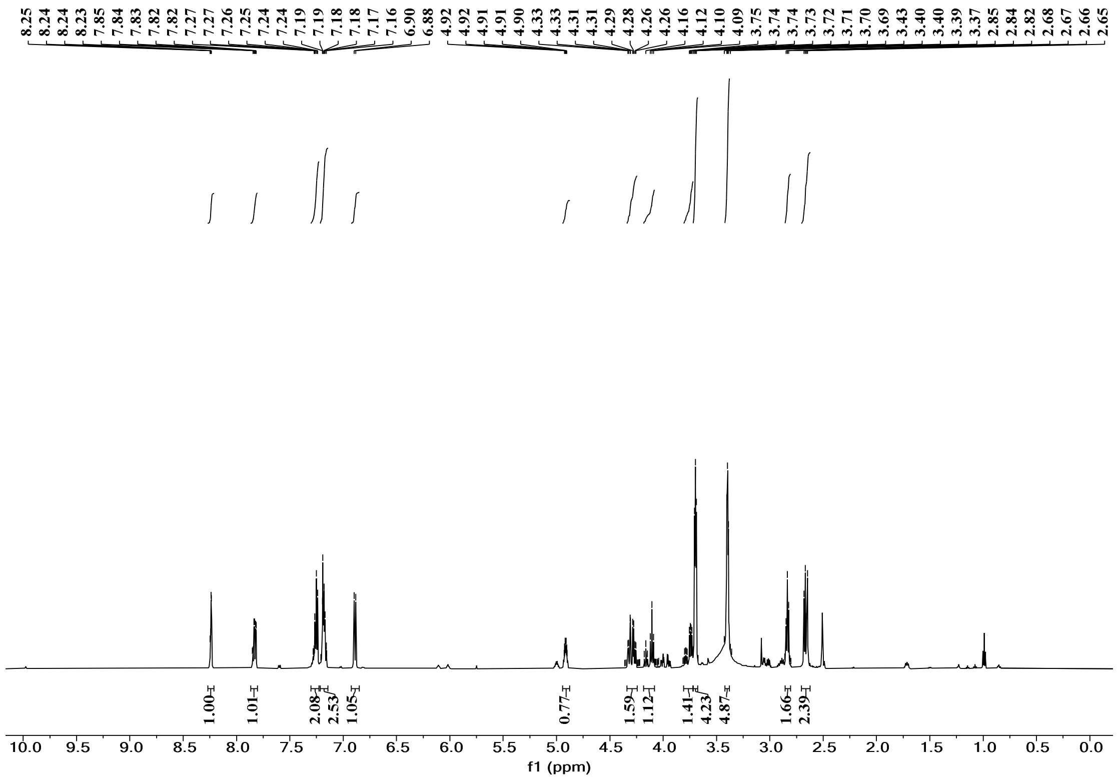

Figure S11. ^{1}H NMR Spectrum (DMSO-d_6, 600 MHz) of **11d**.

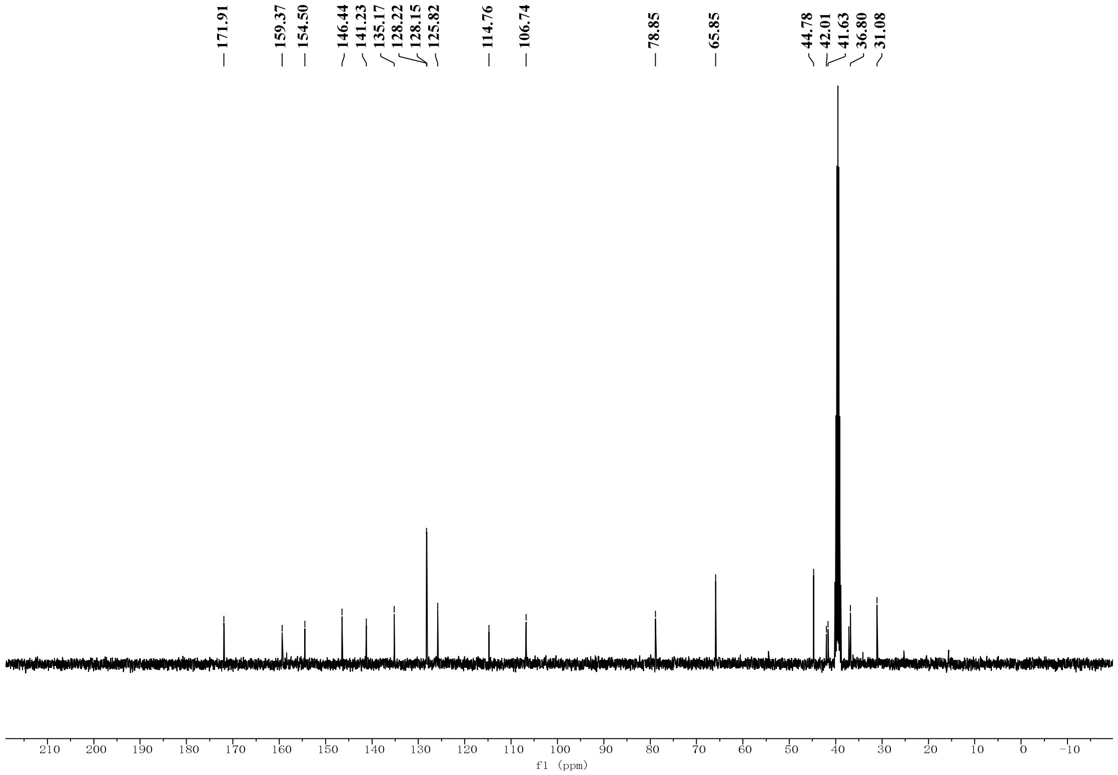

Figure S12. ^{13}C NMR Spectrum (DMSO-d_6, 151 MHz) of **11d**.

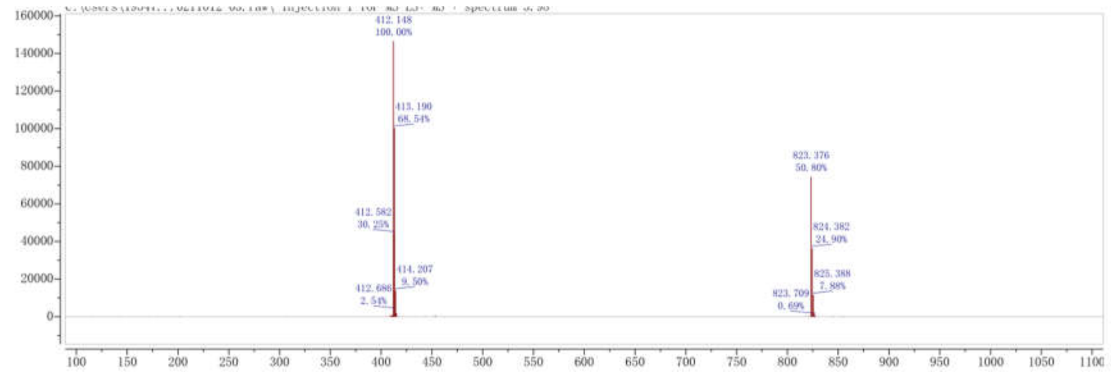

Figure S13. ES-MS for compound **11d**.

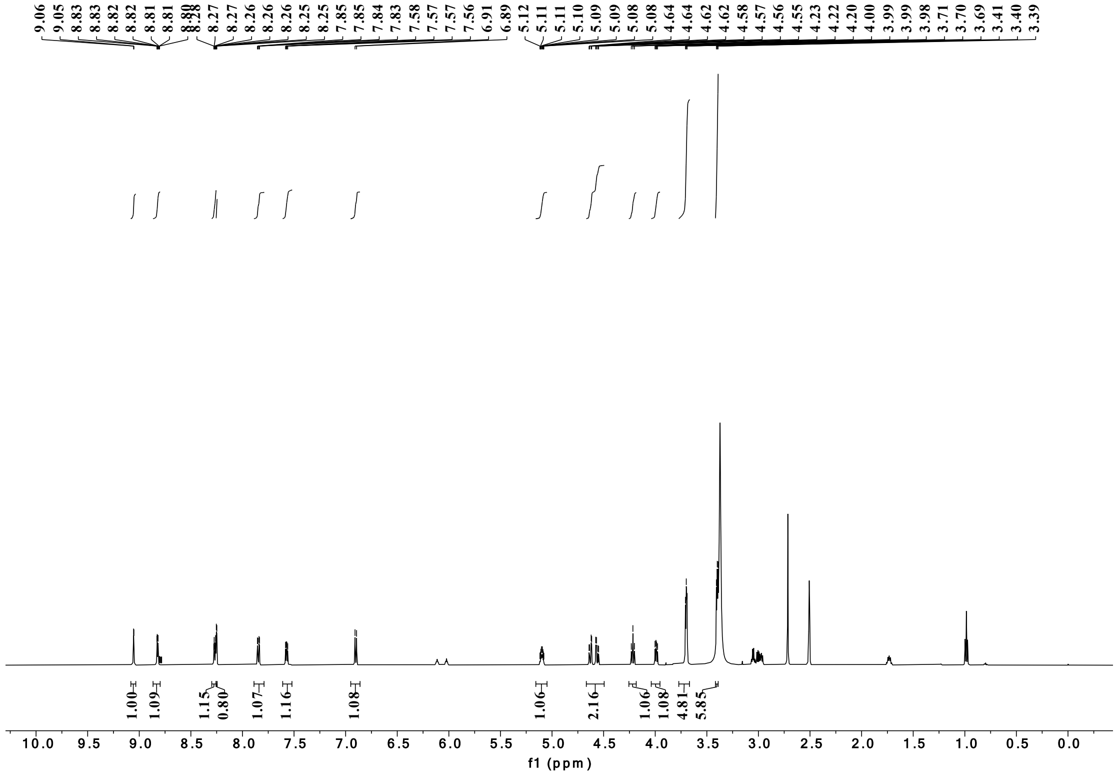

Figure S14. ^{1}H NMR Spectrum (DMSO-d_6, 600 MHz) of **11e**.

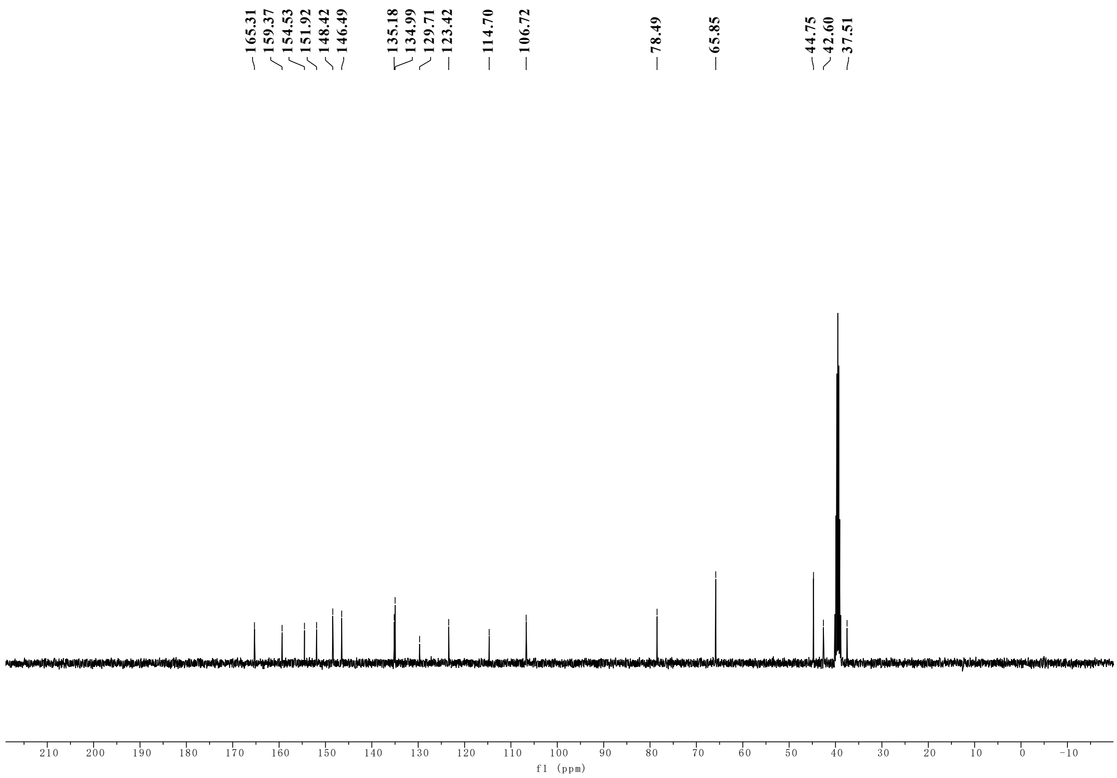

Figure S15. ^{13}C NMR Spectrum (DMSO-d_6, 151 MHz) of **11e**.

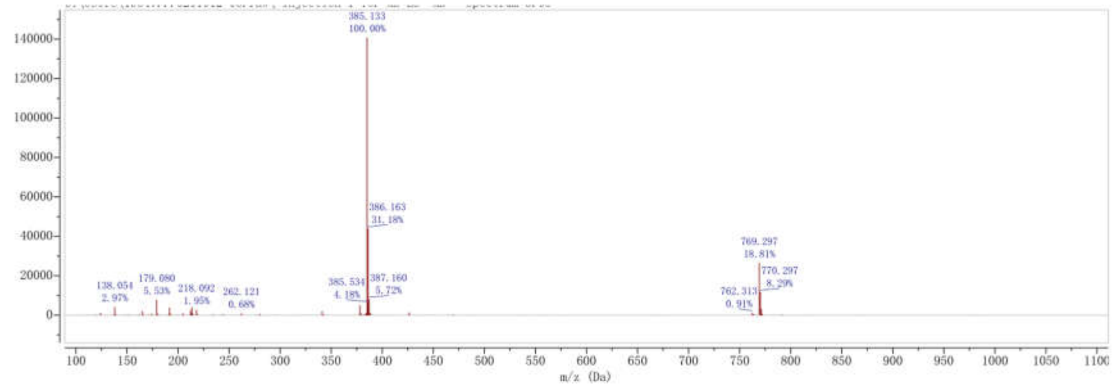

Figure S16. ES-MS for compound **11e**.

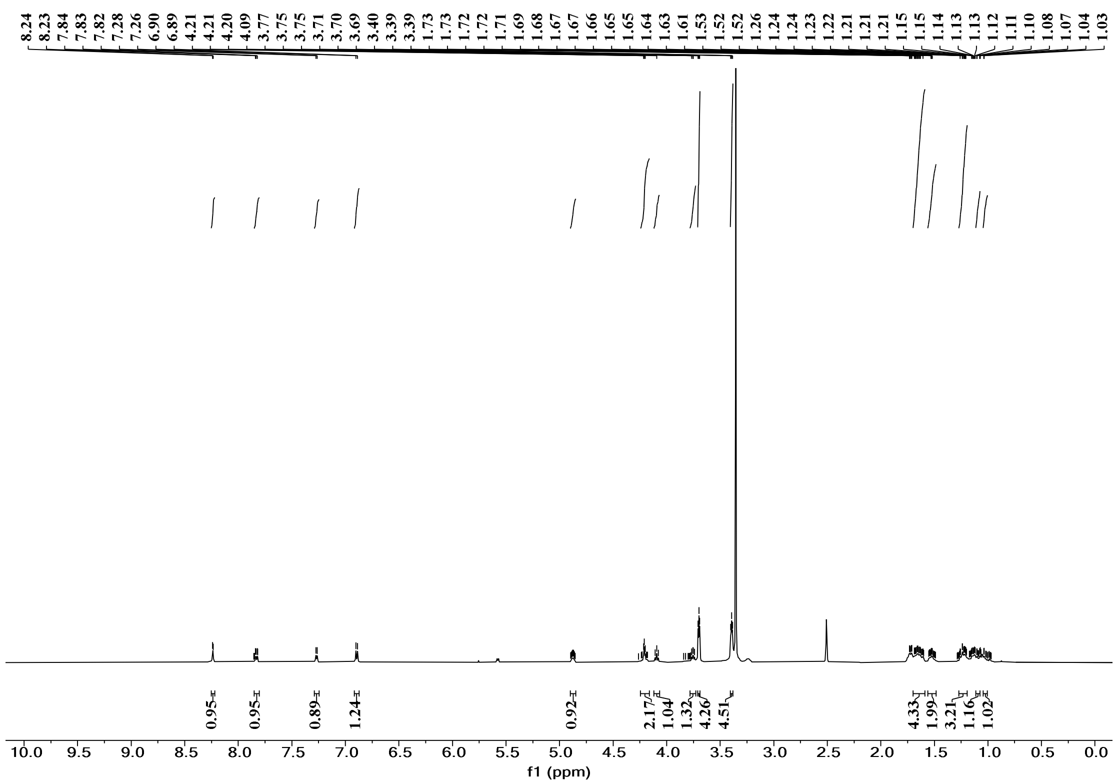

Figure S17. ^{1}H NMR Spectrum (DMSO-d_6, 600 MHz) of **11f**.

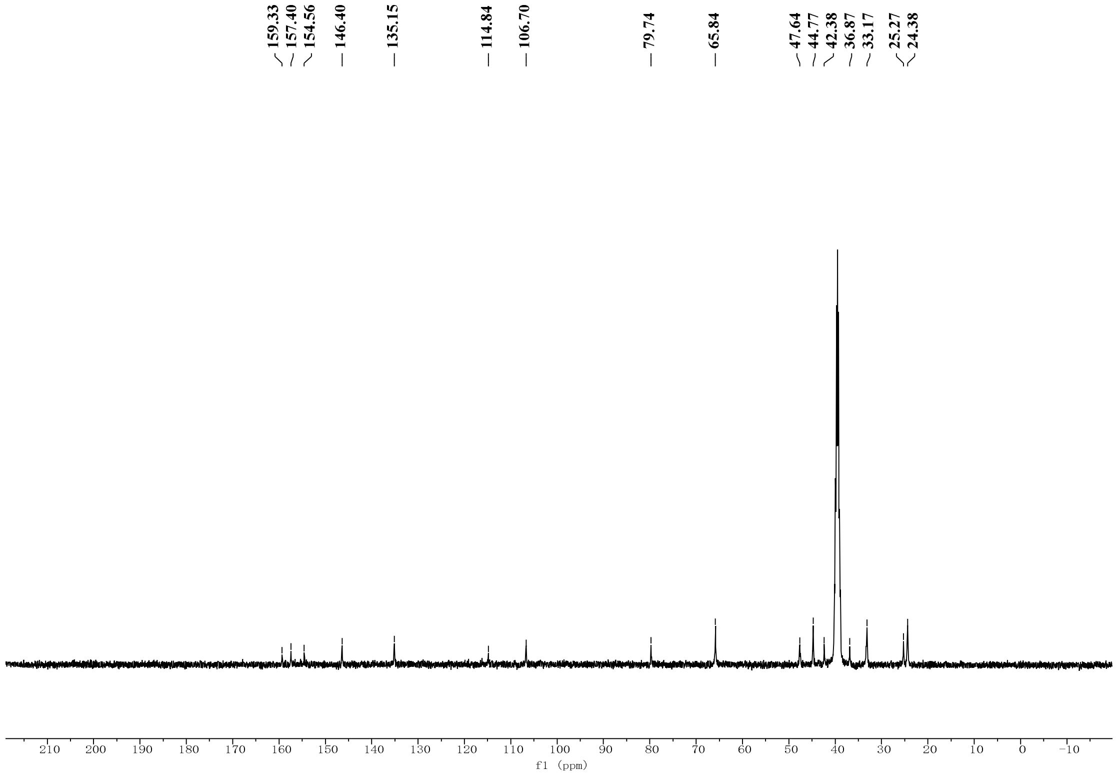

Figure S18. ^{13}C NMR Spectrum (DMSO-d_6, 151 MHz) of **11f**.

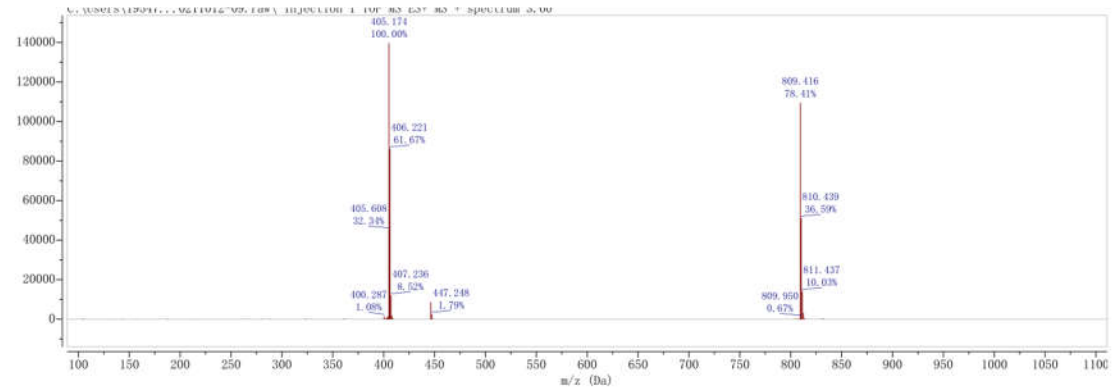

Figure S19. ES-MS for compound **11f**.

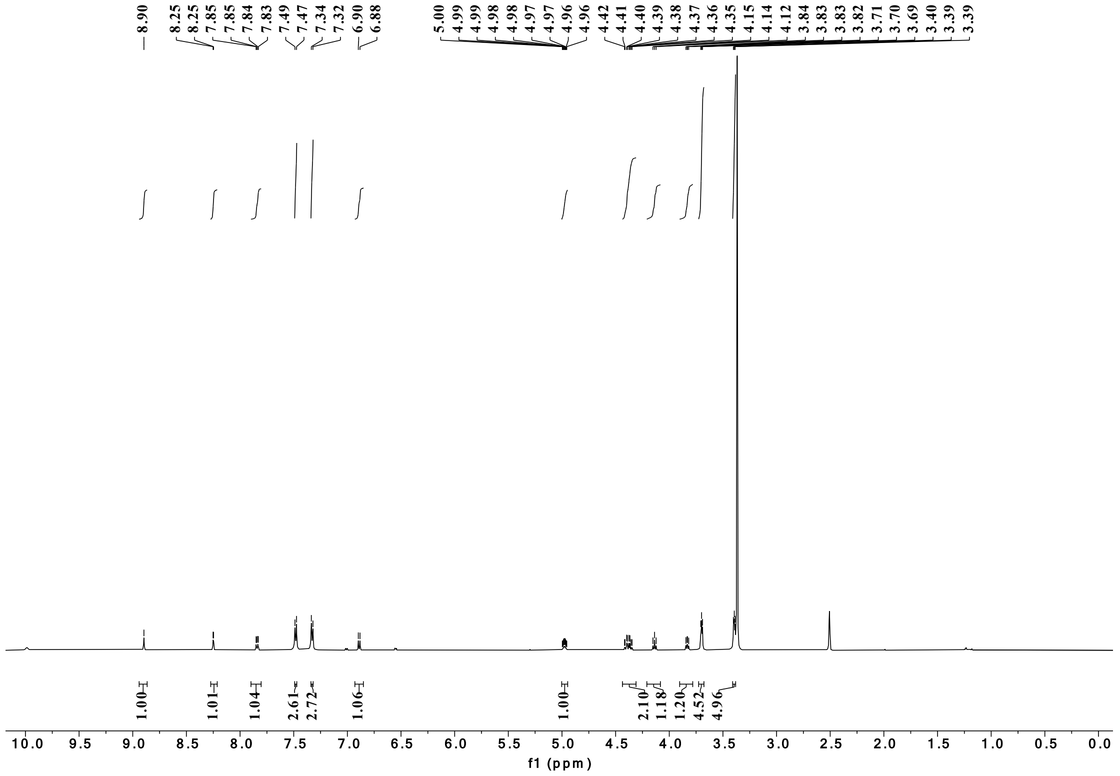

Figure S20. ^{1}H NMR Spectrum (DMSO-d_6, 600 MHz) of **11g**.

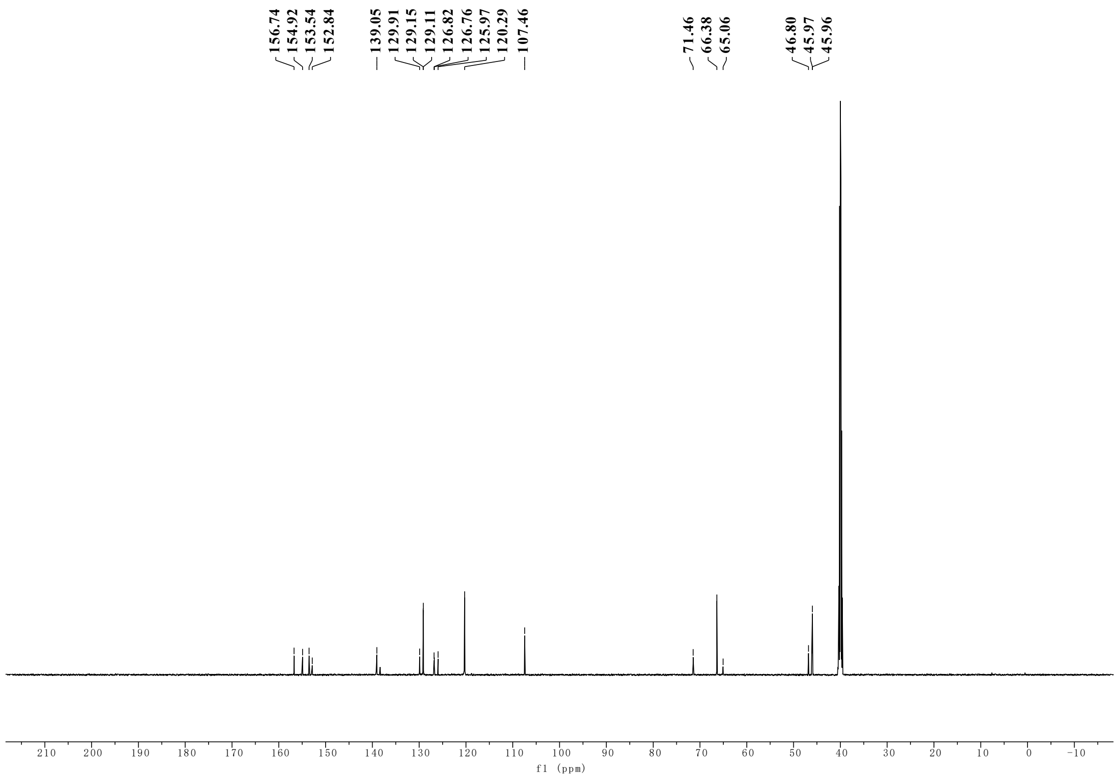

Figure S21. ^{13}C NMR Spectrum (DMSO-d_6, 151 MHz) of **11g**.

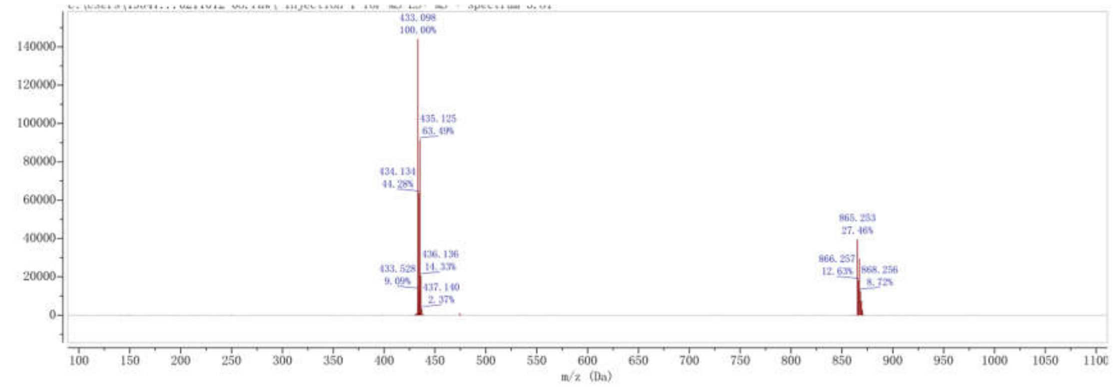

Figure S22. ES-MS for compound **11g**.

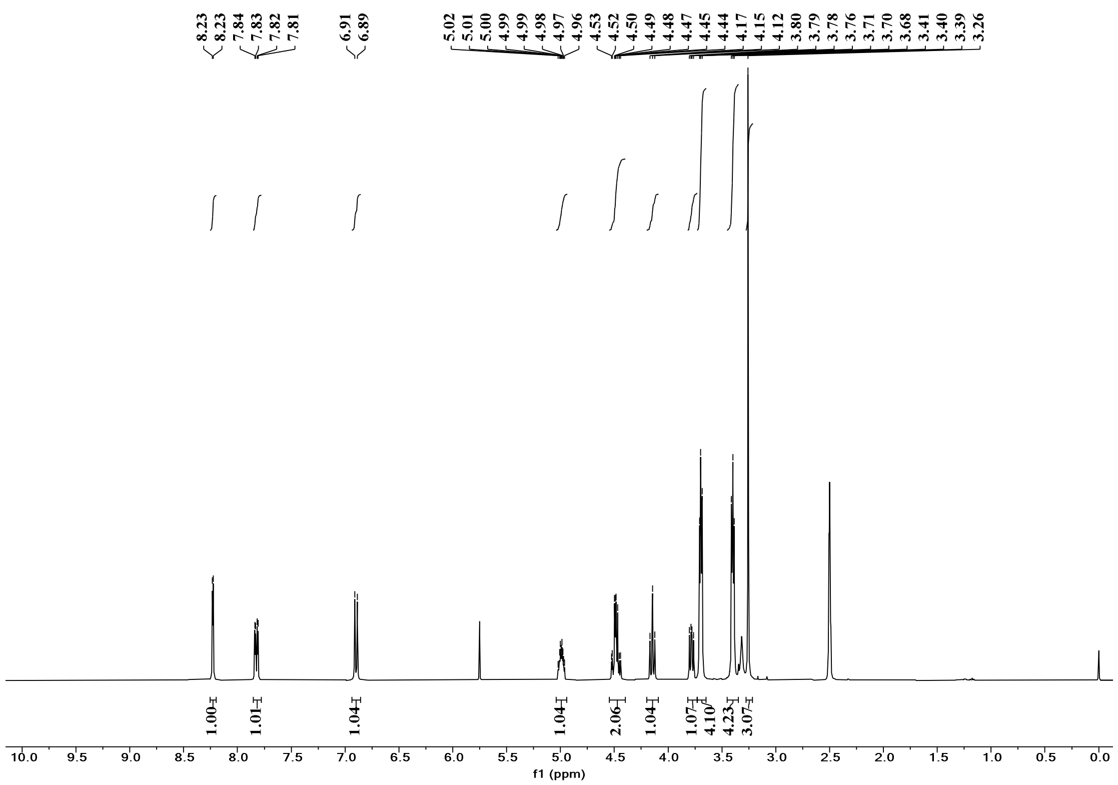

Figure S23. ^{1}H NMR Spectrum (DMSO-d_6, 600 MHz) of **11h**.

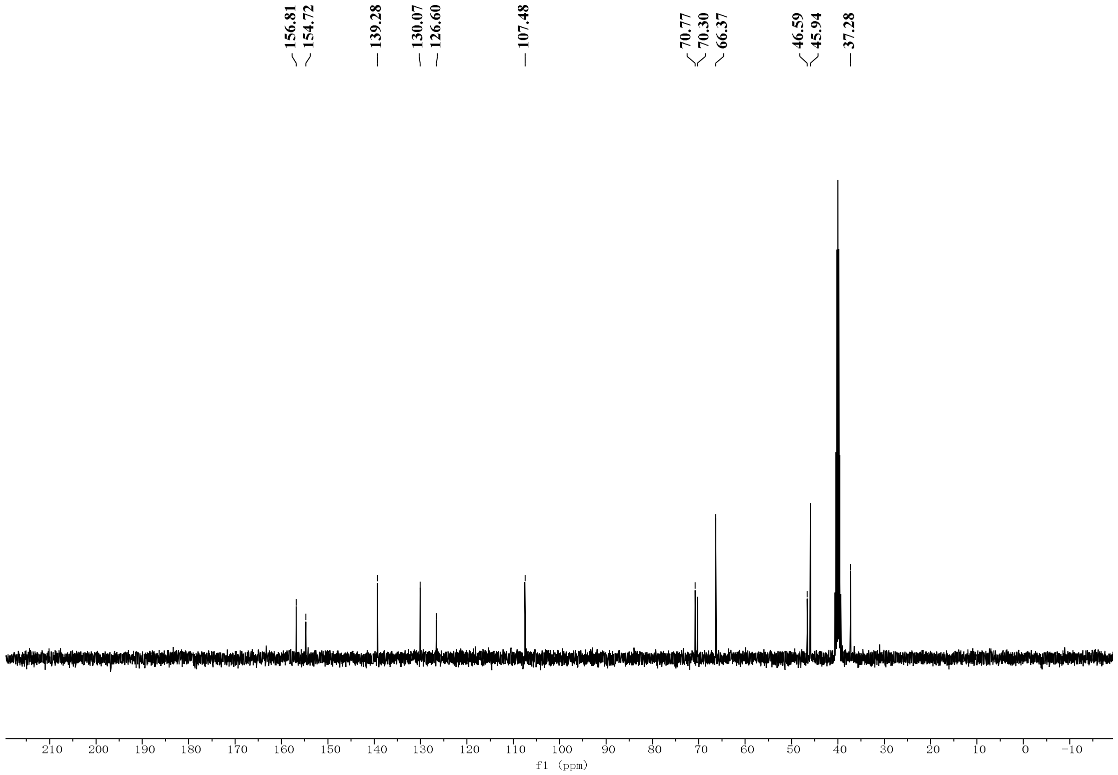

Figure S24. ^{13}C NMR Spectrum (DMSO-d_6, 151 MHz) of **11h**.

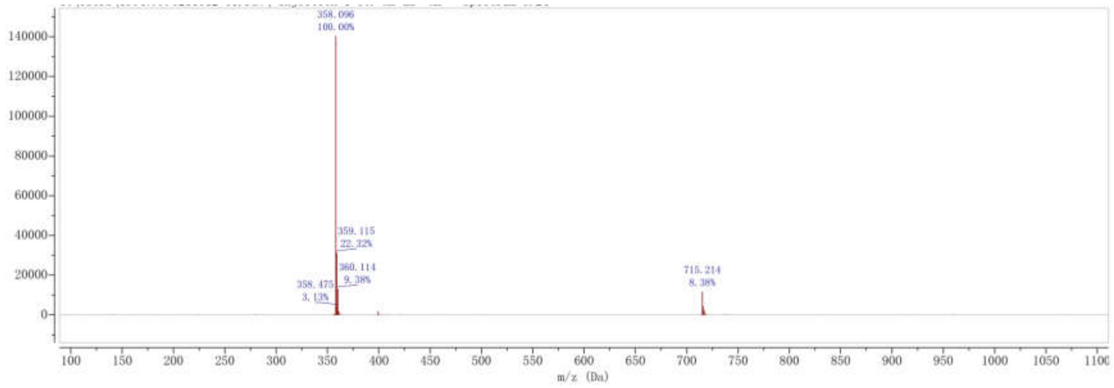

Figure S25. ES-MS for compound **11h**.

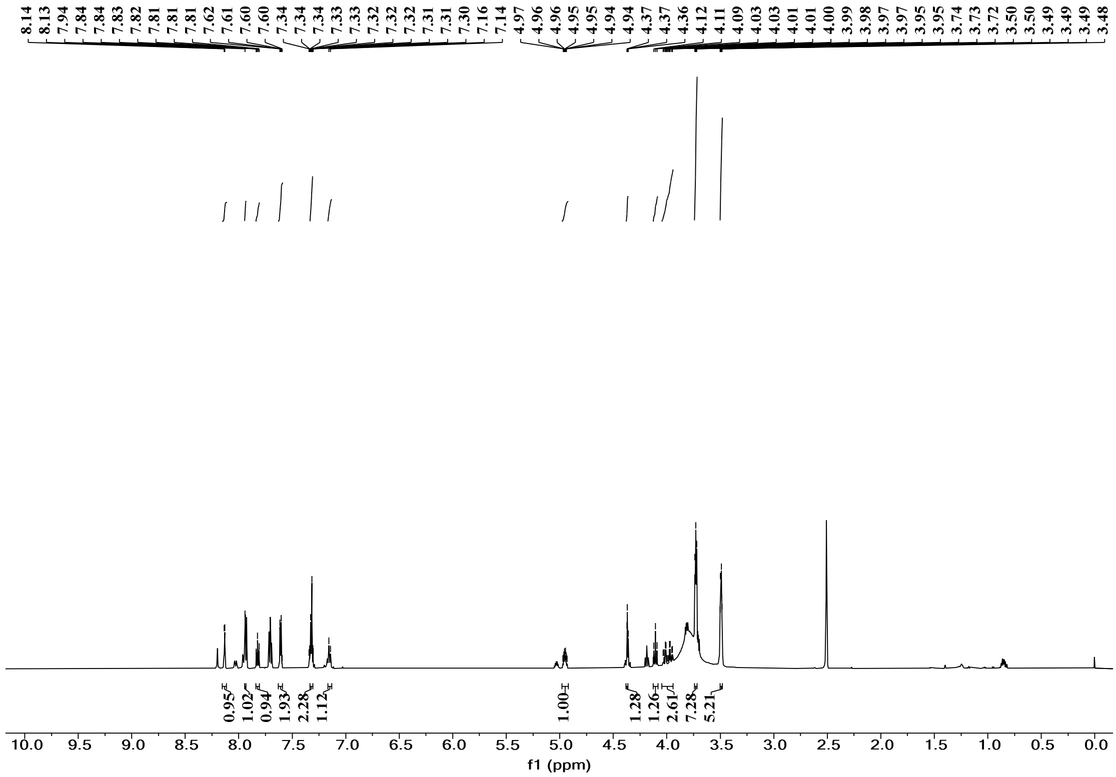

Figure S26. ^{1}H NMR Spectrum (DMSO-d_6, 600 MHz) of **11i**.

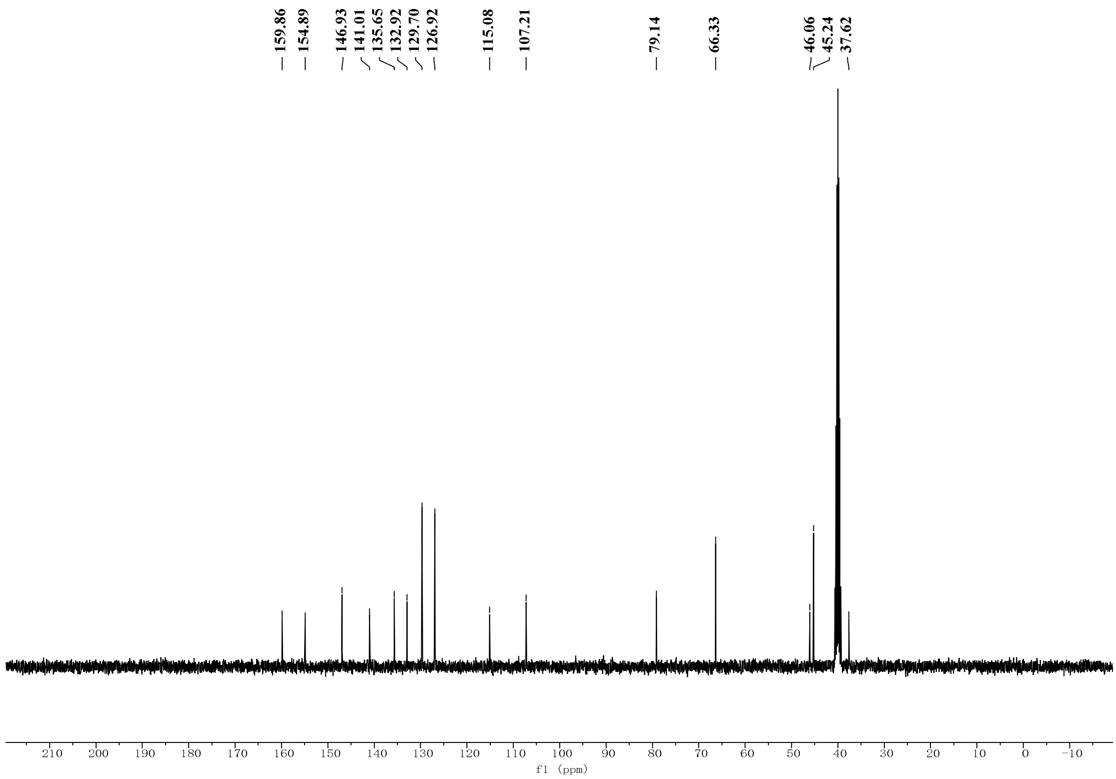

Figure S27. ^{13}C NMR Spectrum (DMSO-d_6, 151 MHz) of **11i**.

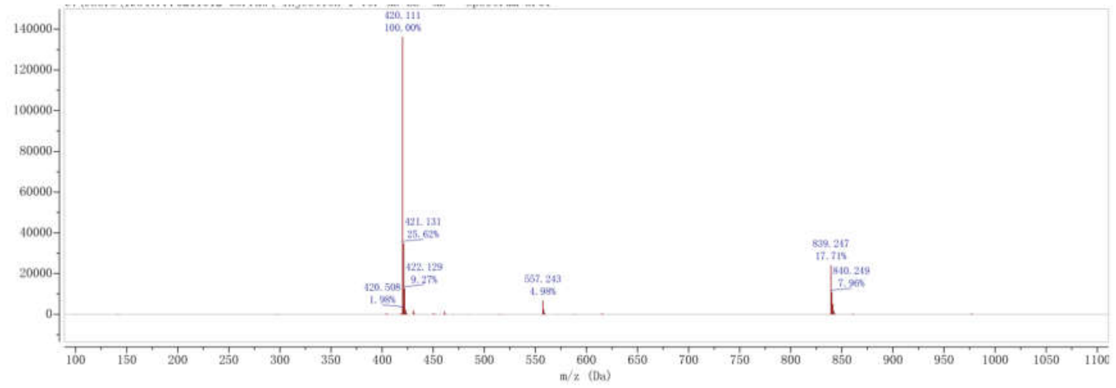

Figure S28. ES-MS for compound **11i**.

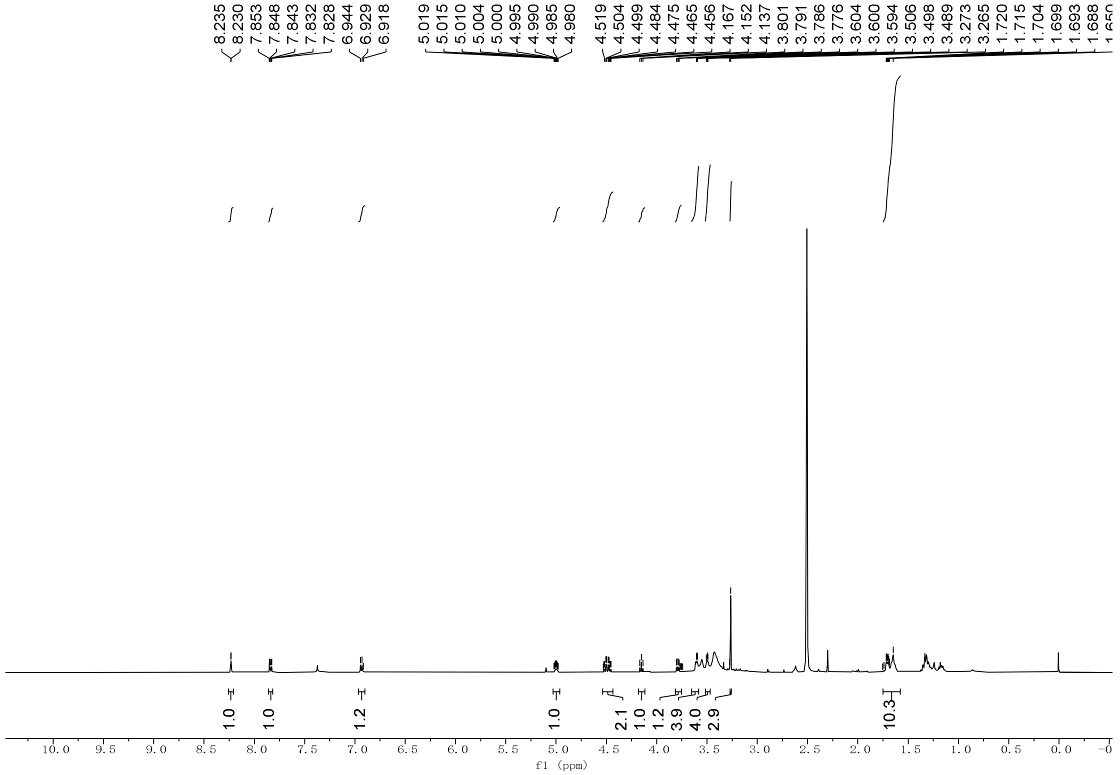

Figure S29. ^{1}H NMR Spectrum (DMSO-d_6, 600 MHz) of **12a**.

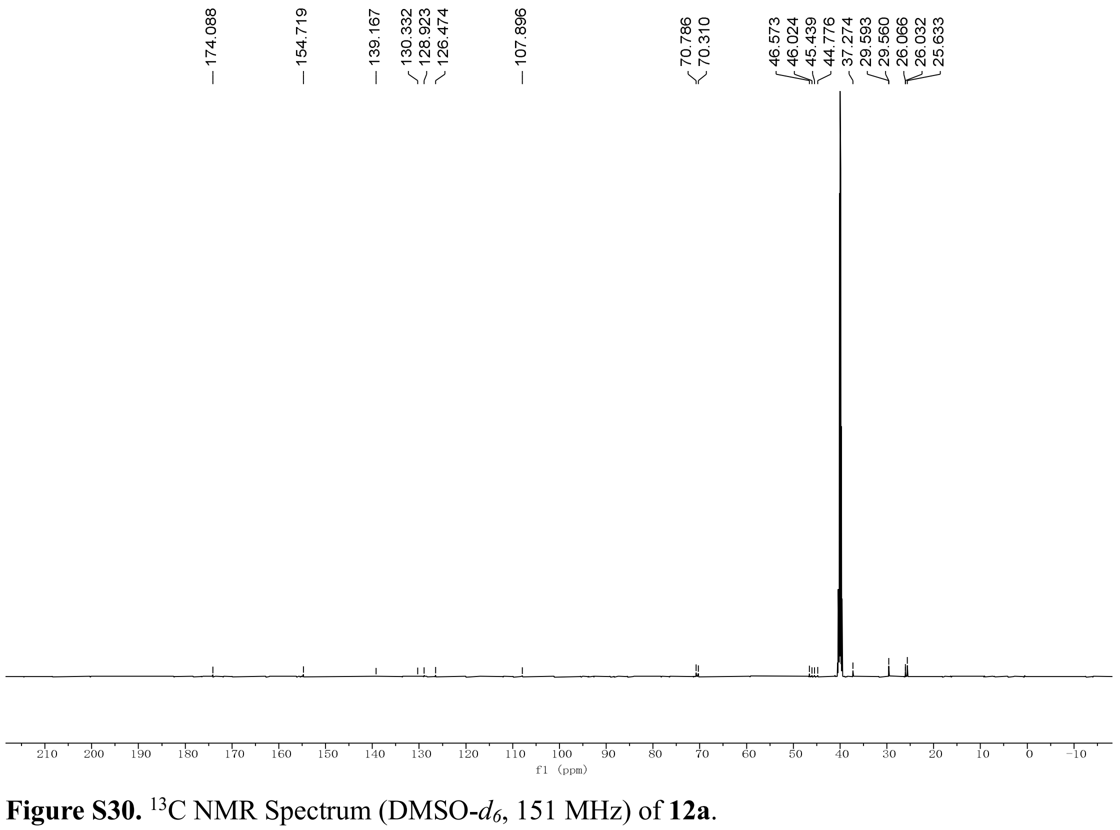

Figure S30. ^{13}C NMR Spectrum (DMSO-d_6, 151 MHz) of **12a**.

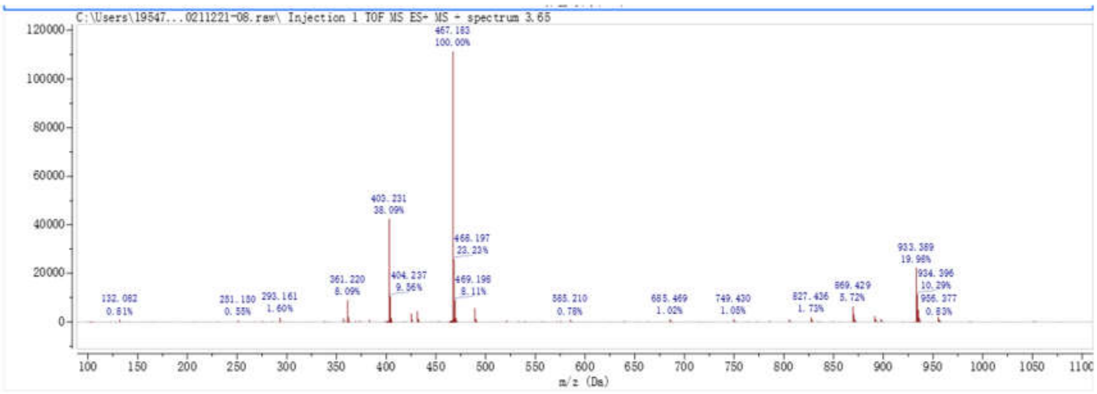

Figure S31. ES-MS for compound **12a.**

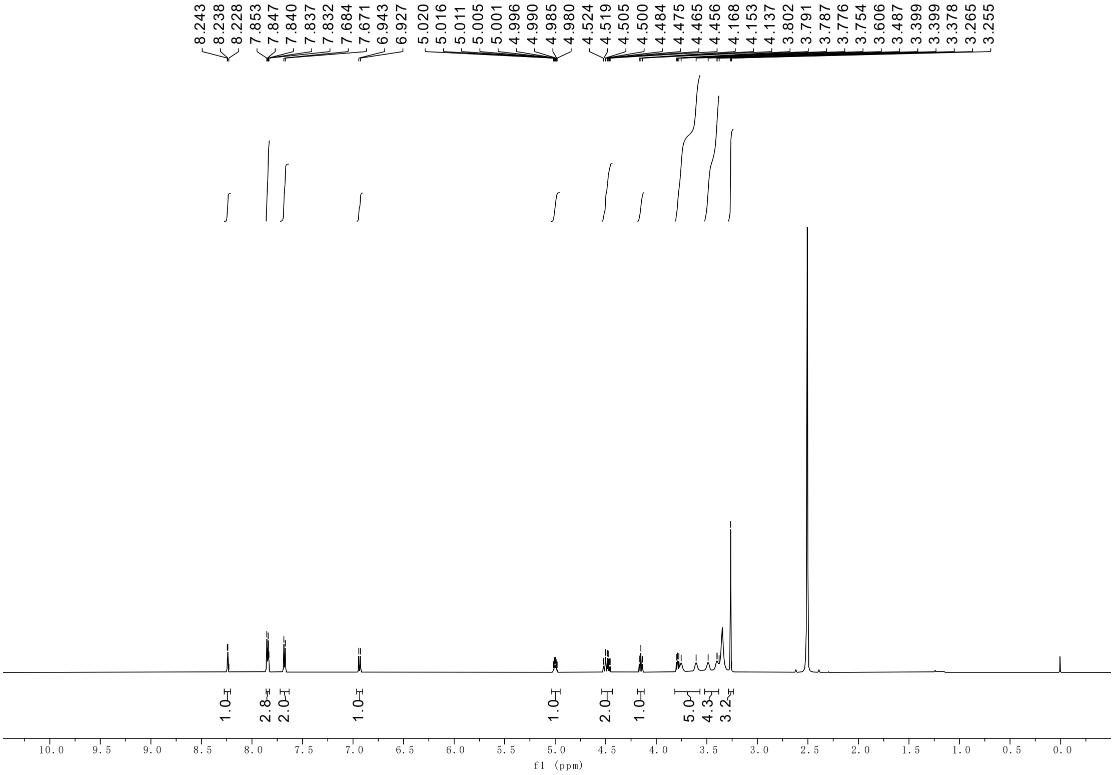

Figure S32. ^{1}H NMR Spectrum (DMSO-d_6, 600 MHz) of **12b**.

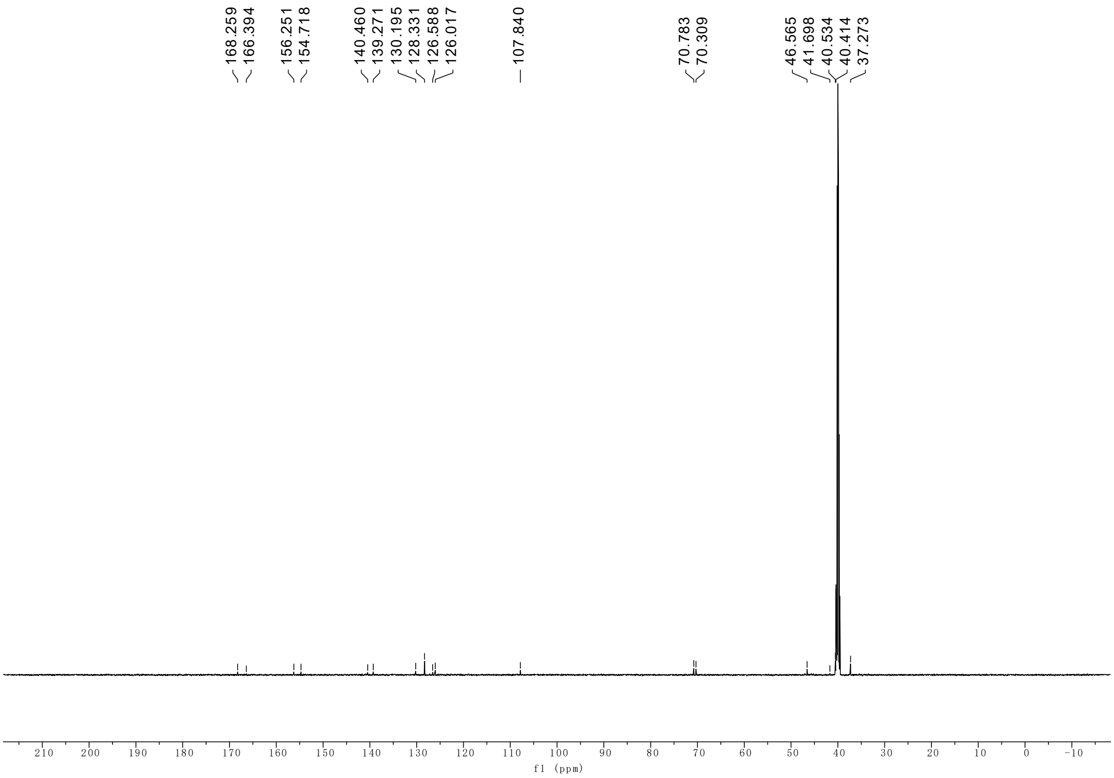

Figure S33. ^{13}C NMR Spectrum (DMSO-d_6, 151 MHz) of **12b**.

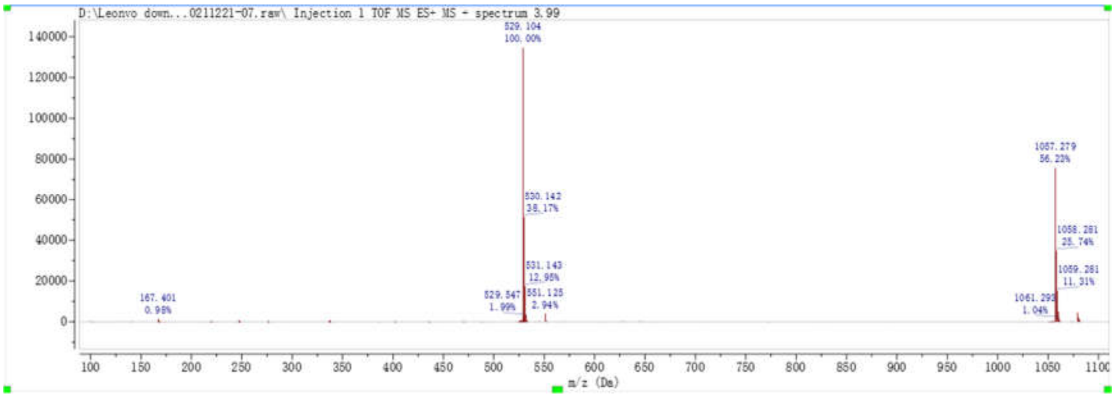

Figure S34. ES-MS for compound **12b.**

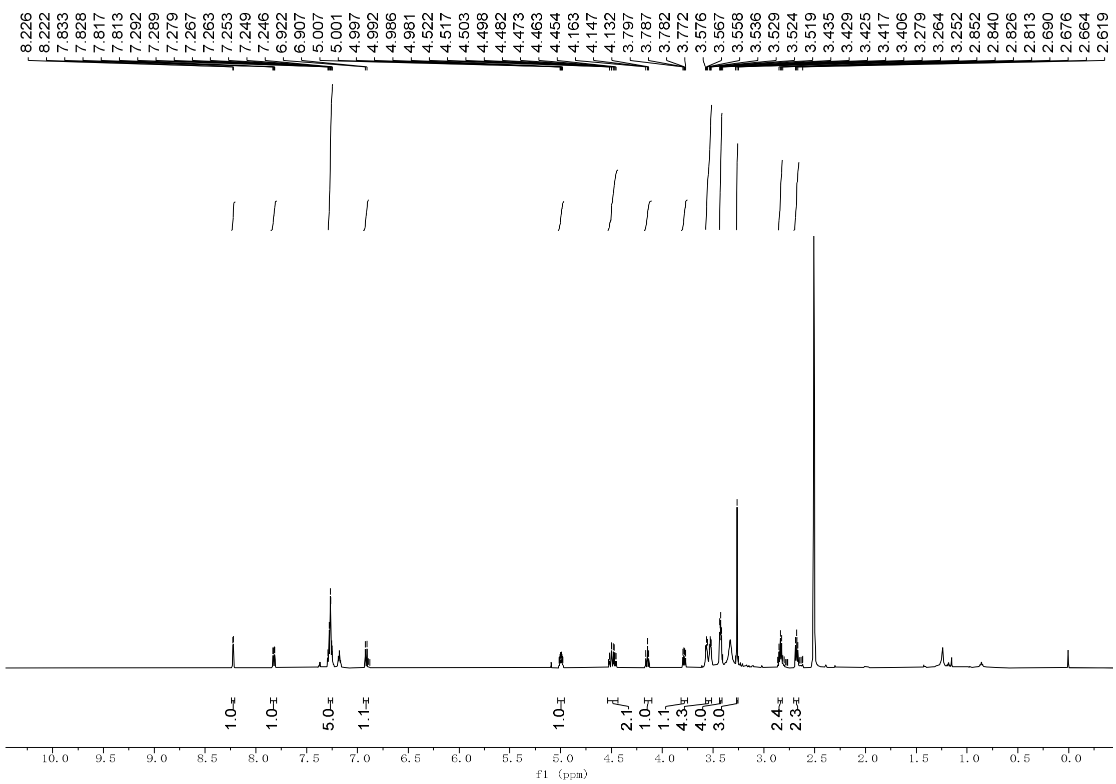

Figure S35. ^{1}H NMR Spectrum (DMSO-d_6, 600 MHz) of **12c**.

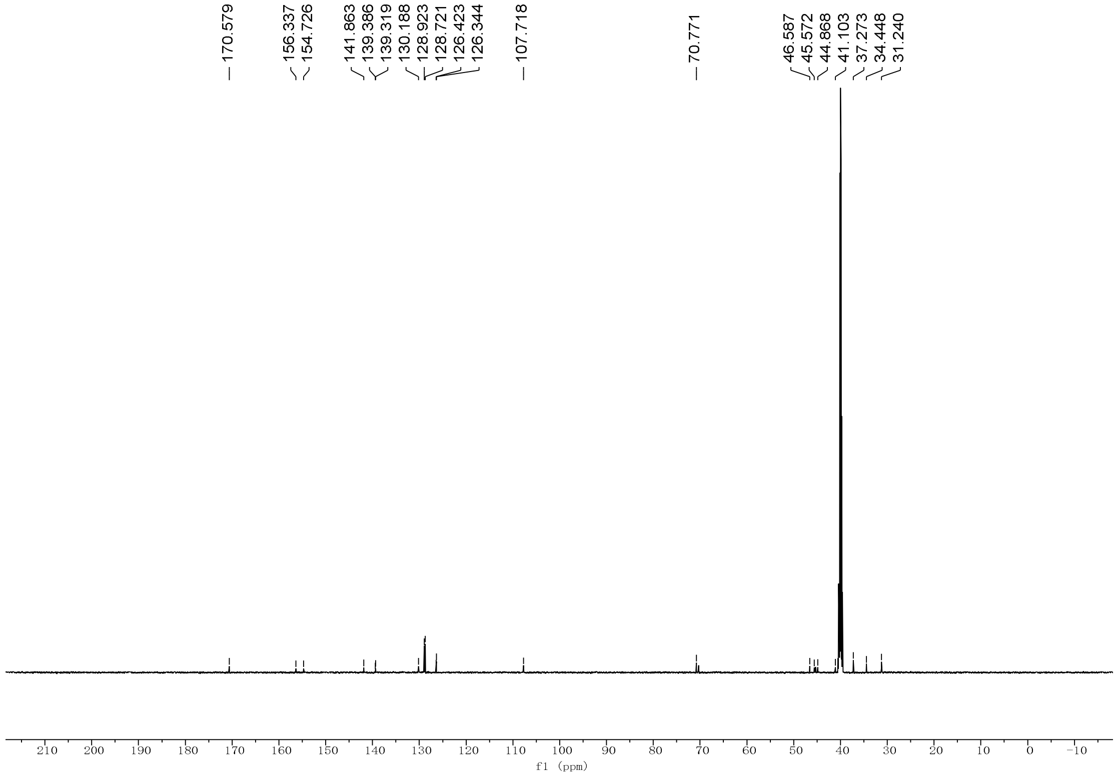

Figure S36. ^{13}C NMR Spectrum (DMSO-d_6, 151 MHz) of **12c**.

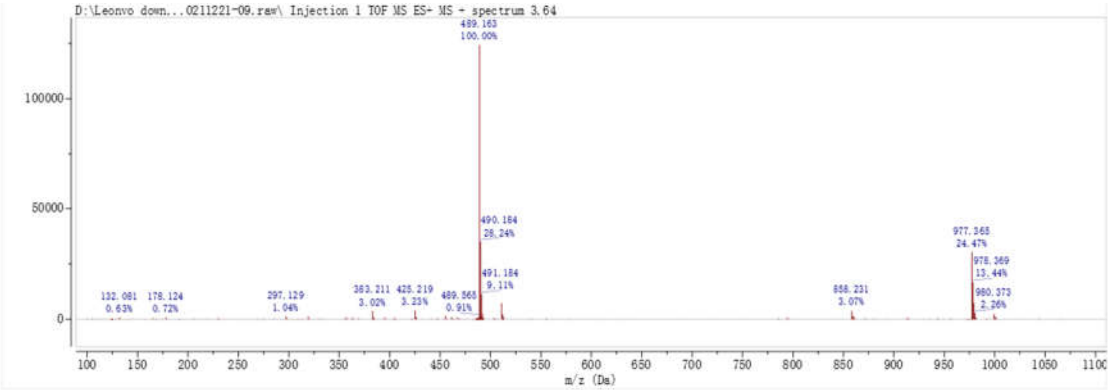

Figure S37. ES-MS for compound **12c.**

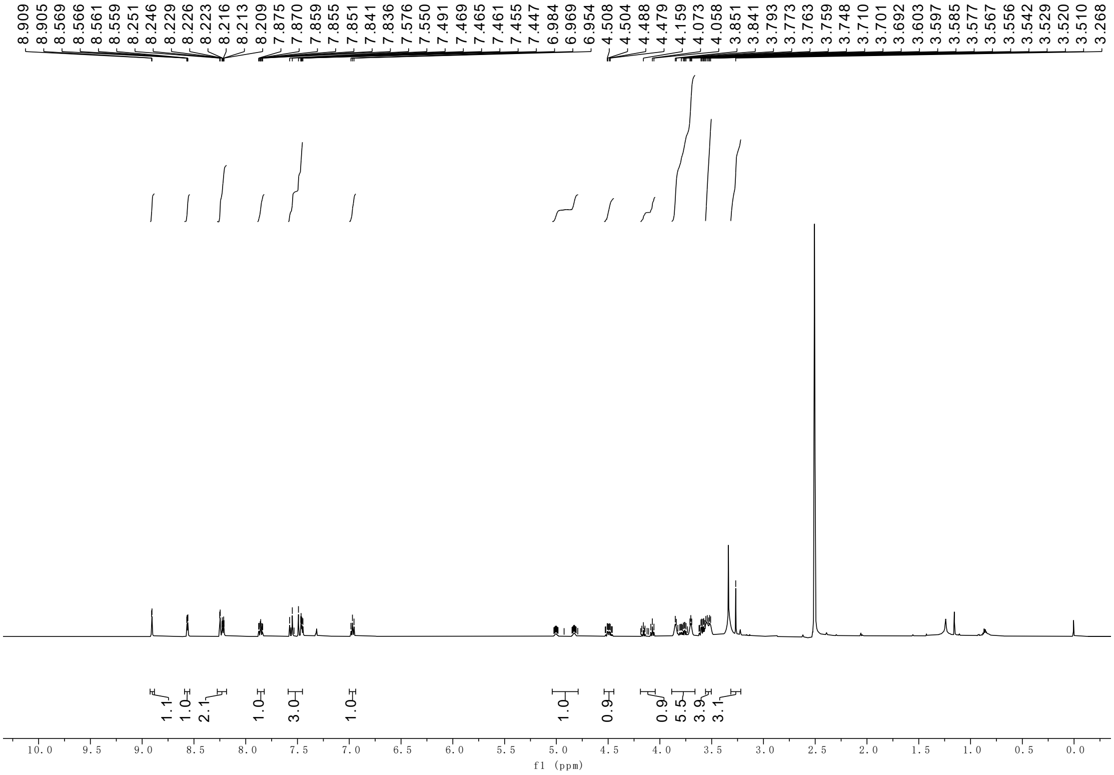

Figure S38. ^{1}H NMR Spectrum (DMSO-d_6, 600 MHz) of **12d**.

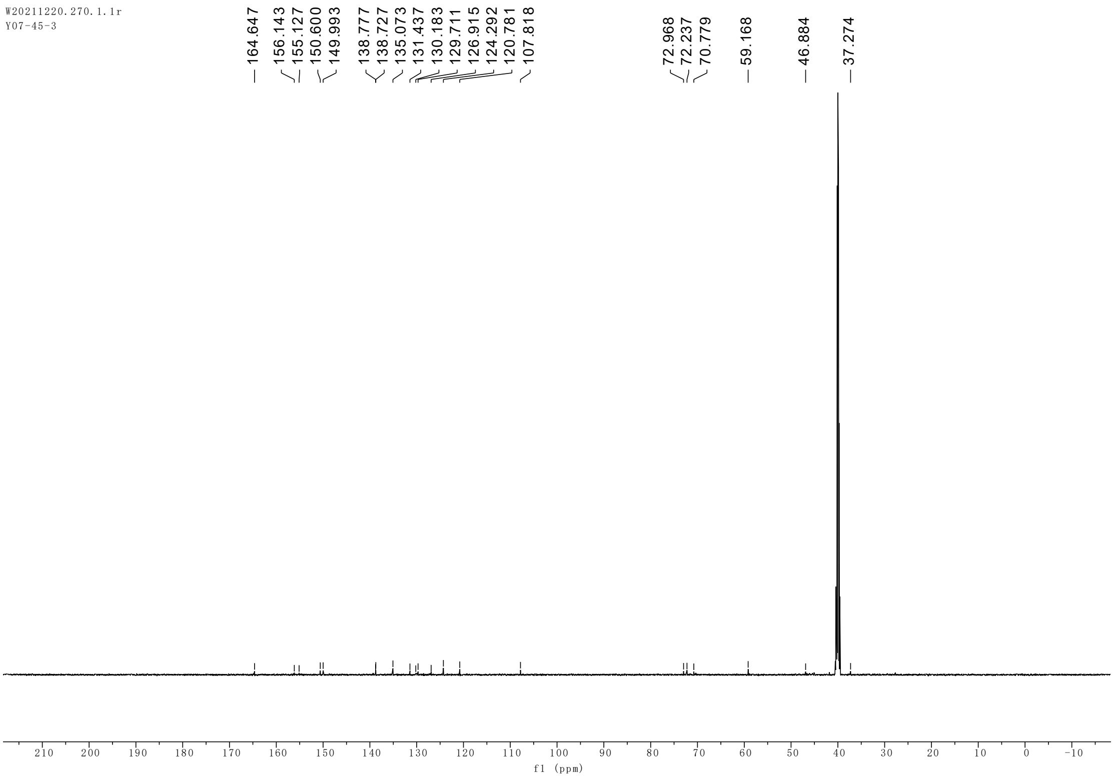

Figure S39. ^{13}C NMR Spectrum (DMSO-d_6, 151 MHz) of **12d**.

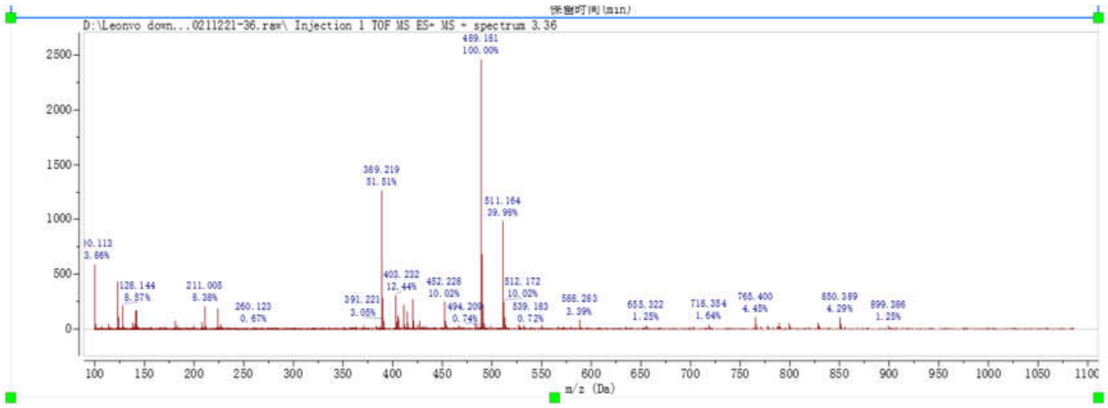

Figure S40. ES-MS for compound **12d.**

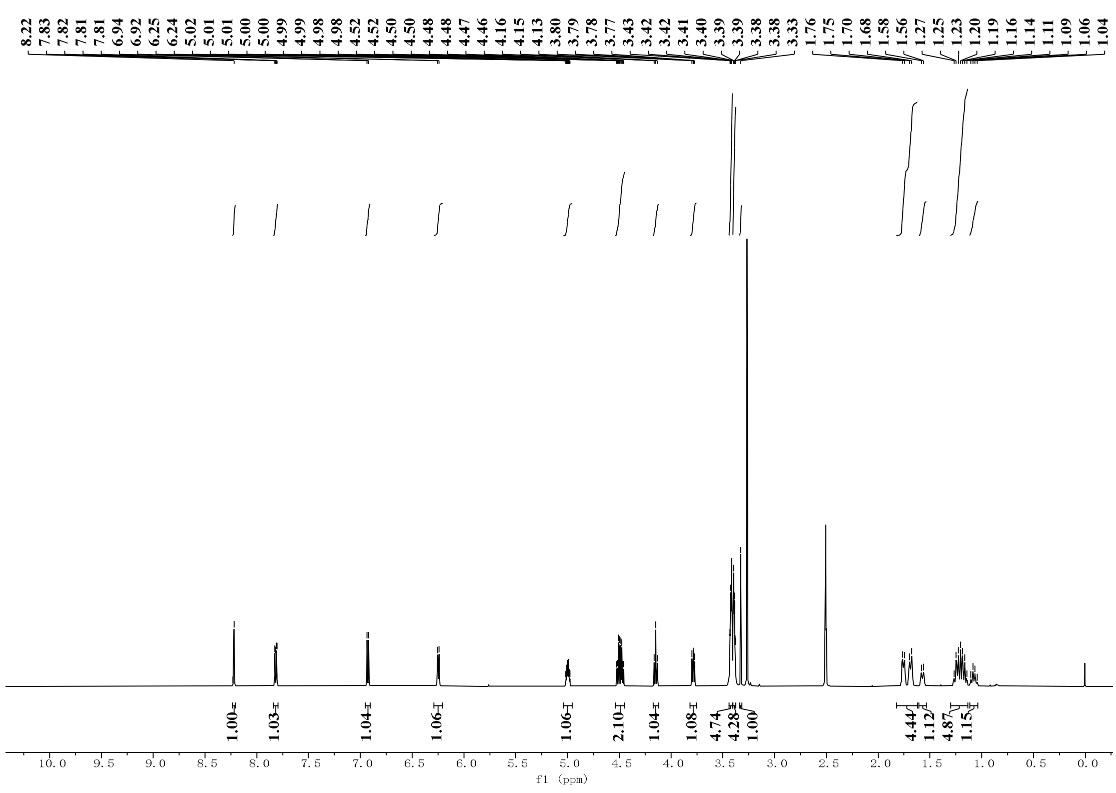

Figure S41. ^{1}H NMR Spectrum (DMSO-d_6, 600 MHz) of **12e**.

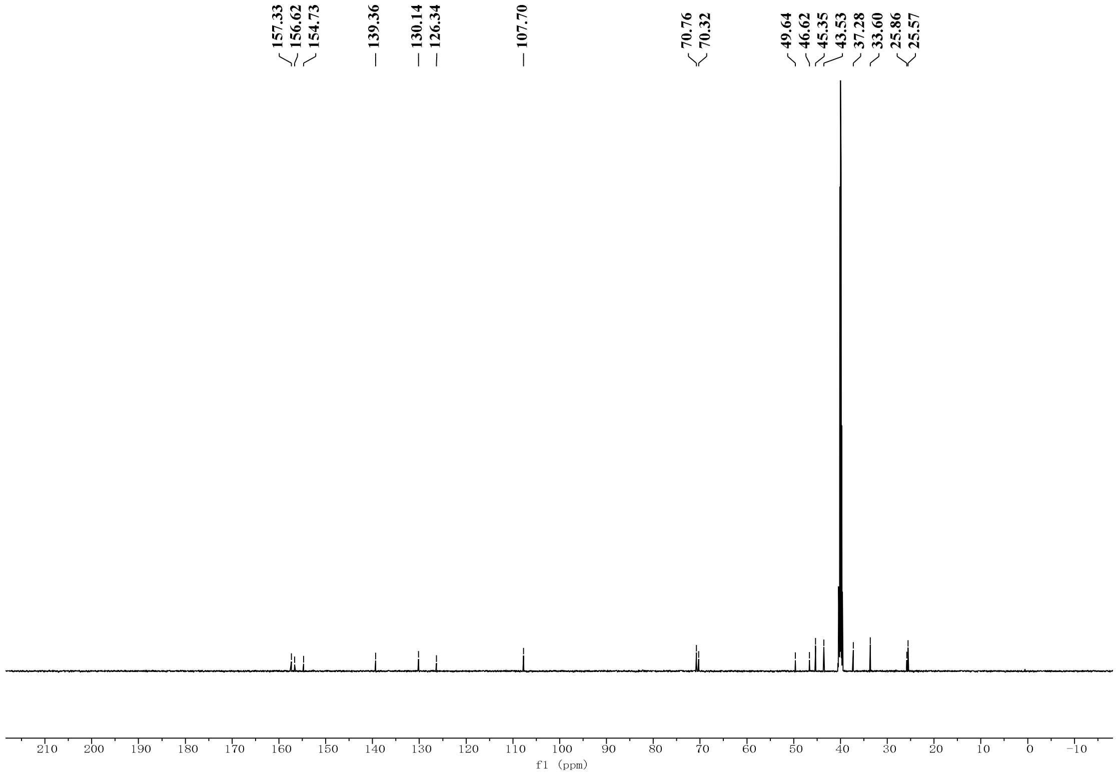

Figure S42. ^{13}C NMR Spectrum (DMSO-d_6, 151 MHz) of **12e**.

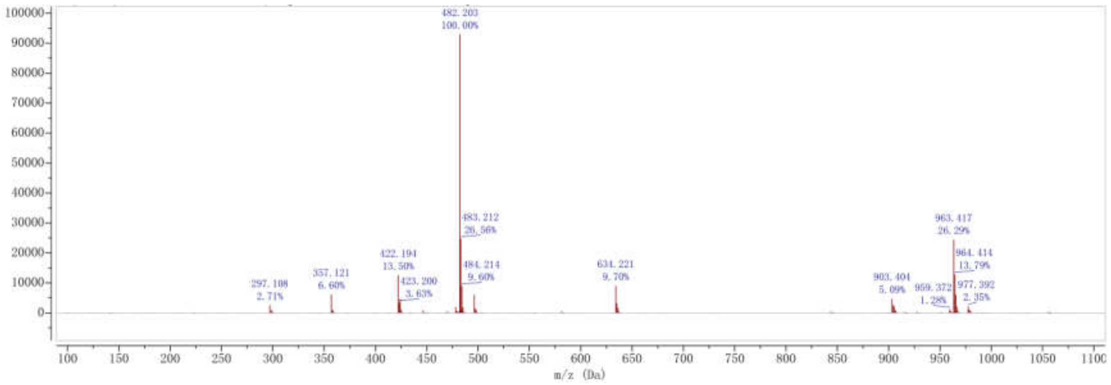

Figure S43. ES-MS for compound **12e.**

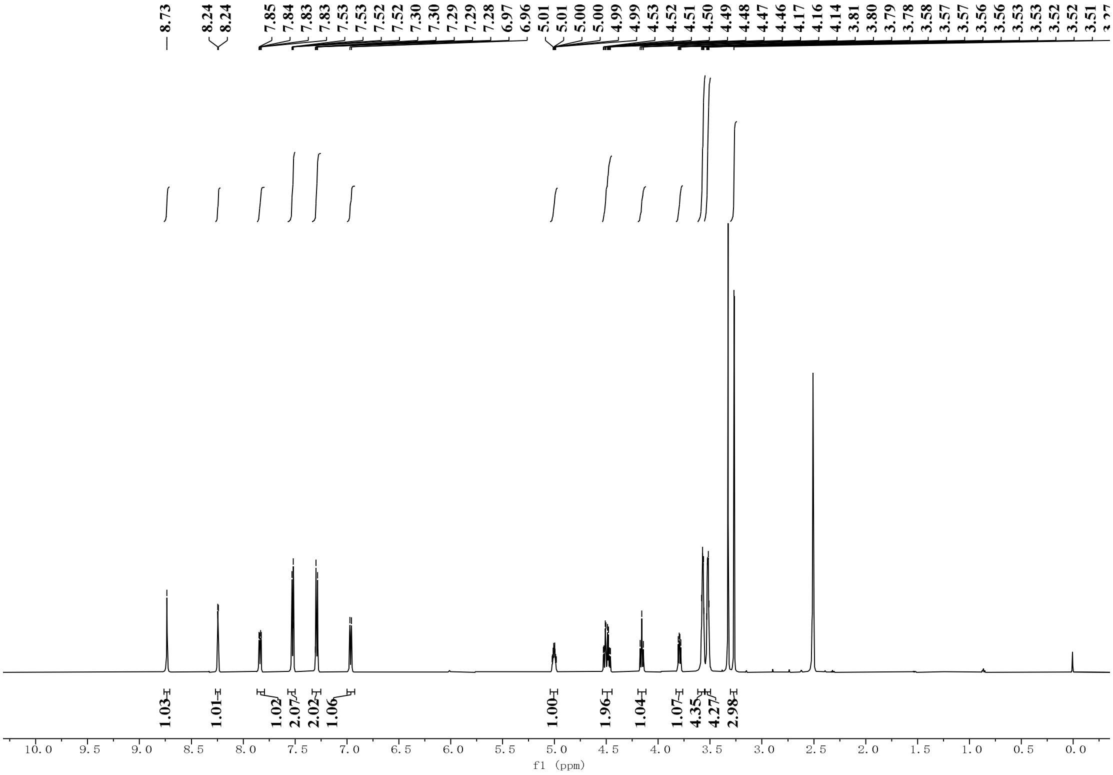

Figure S44. ^{1}H NMR Spectrum (DMSO-d_6, 600 MHz) of **12f**.

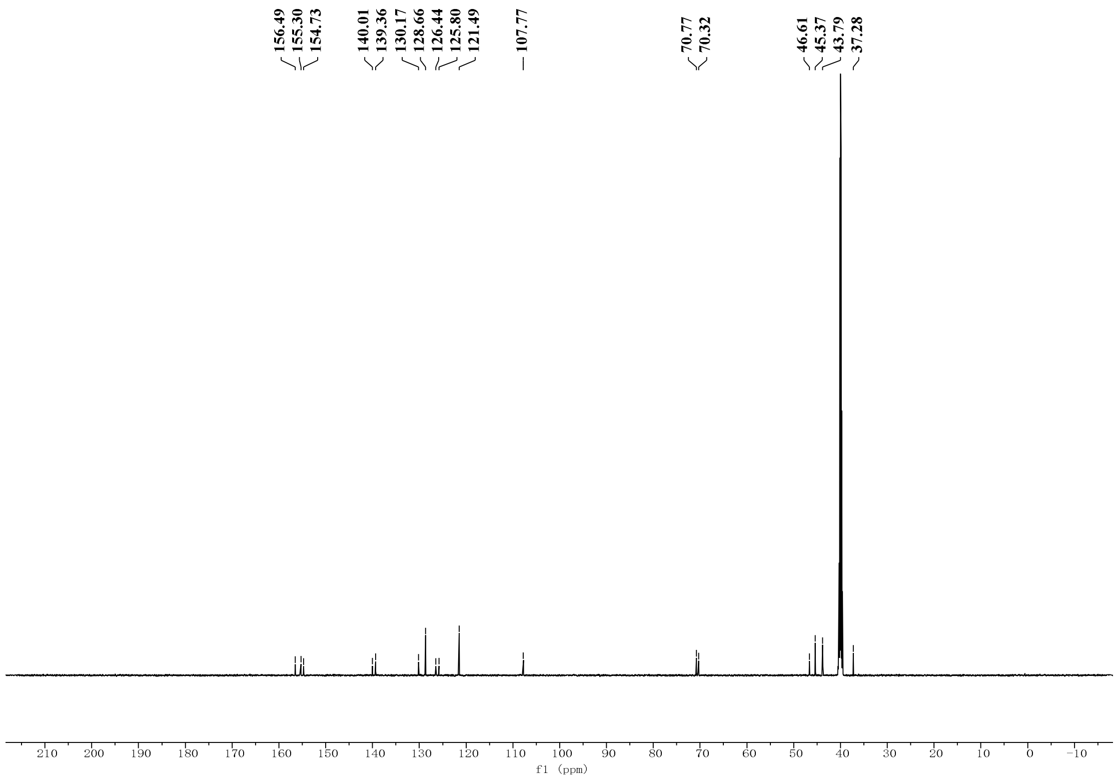

Figure S45. ^{13}C NMR Spectrum (DMSO-d_6, 151 MHz) of **12f**.

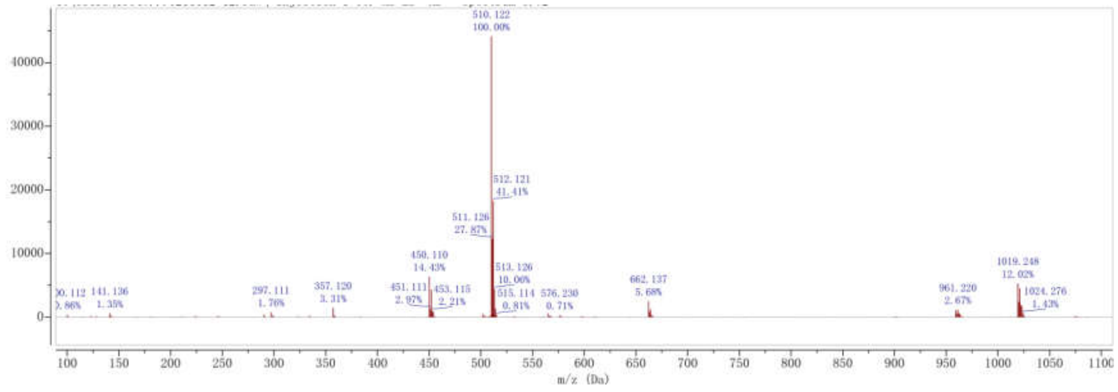

Figure S46. ES-MS for compound **12f.**

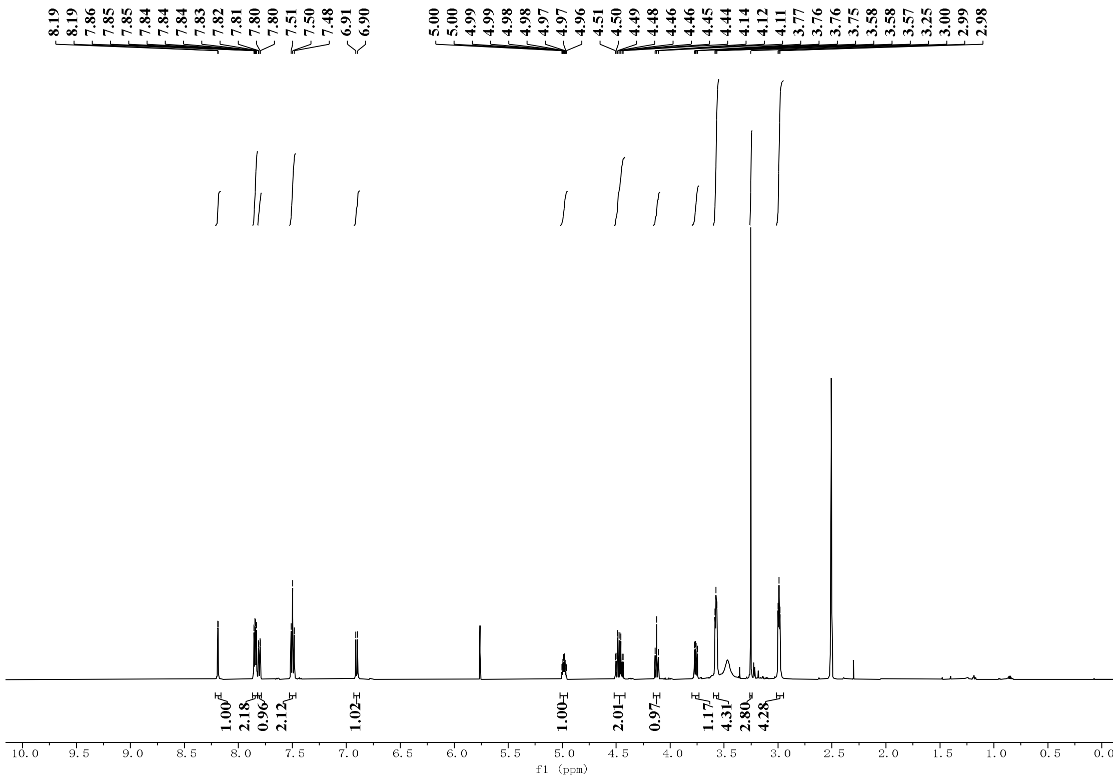

Figure S47. ^{1}H NMR Spectrum (DMSO-d_6, 600 MHz) of **12g**.

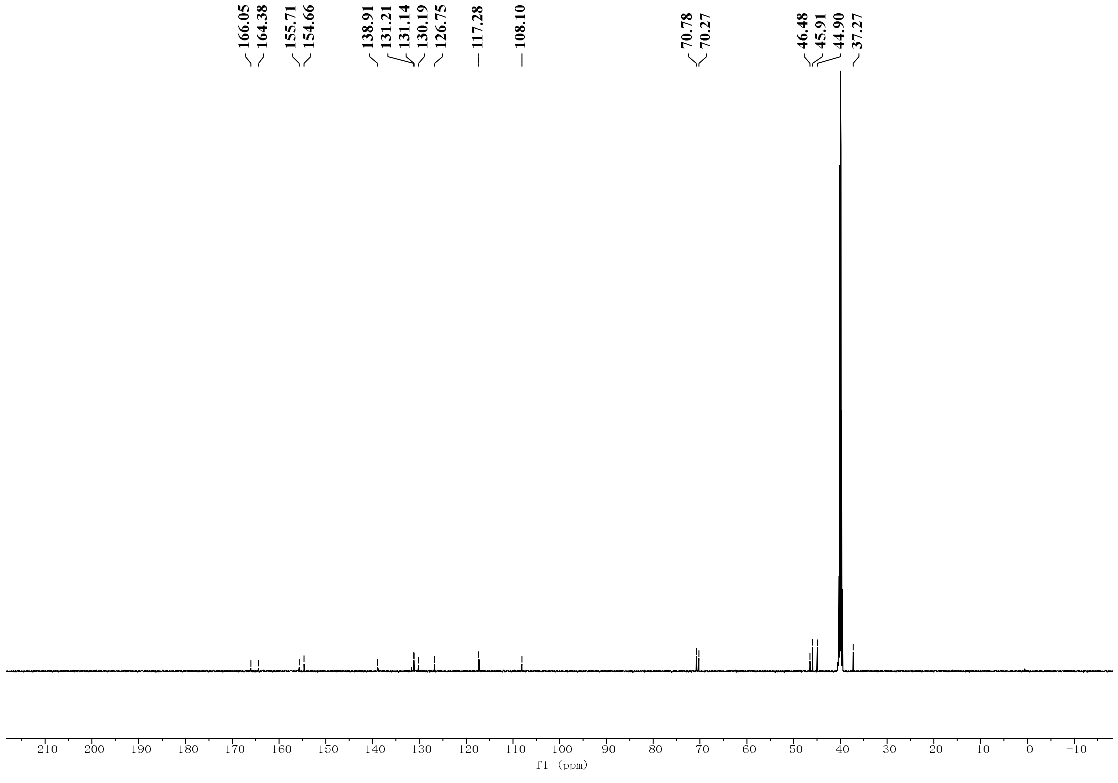

Figure S48. ^{13}C NMR Spectrum (DMSO-d_6, 151 MHz) of **12g**.

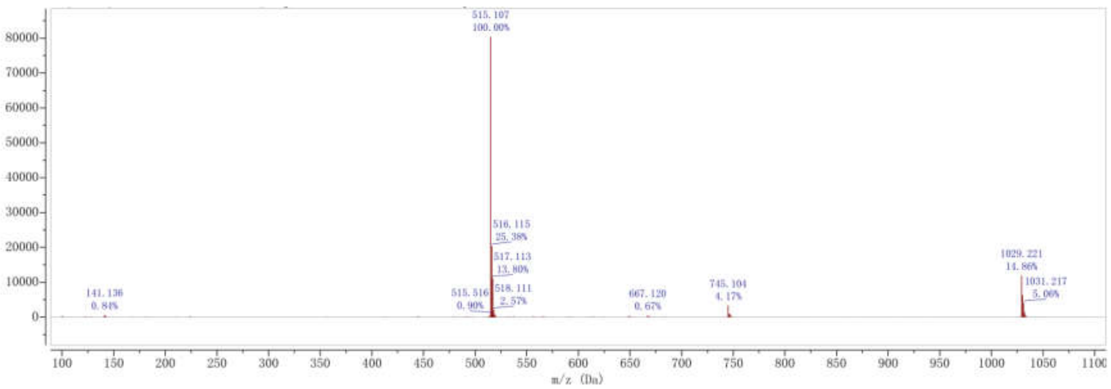

Figure S49. ES-MS for compound **12g.**

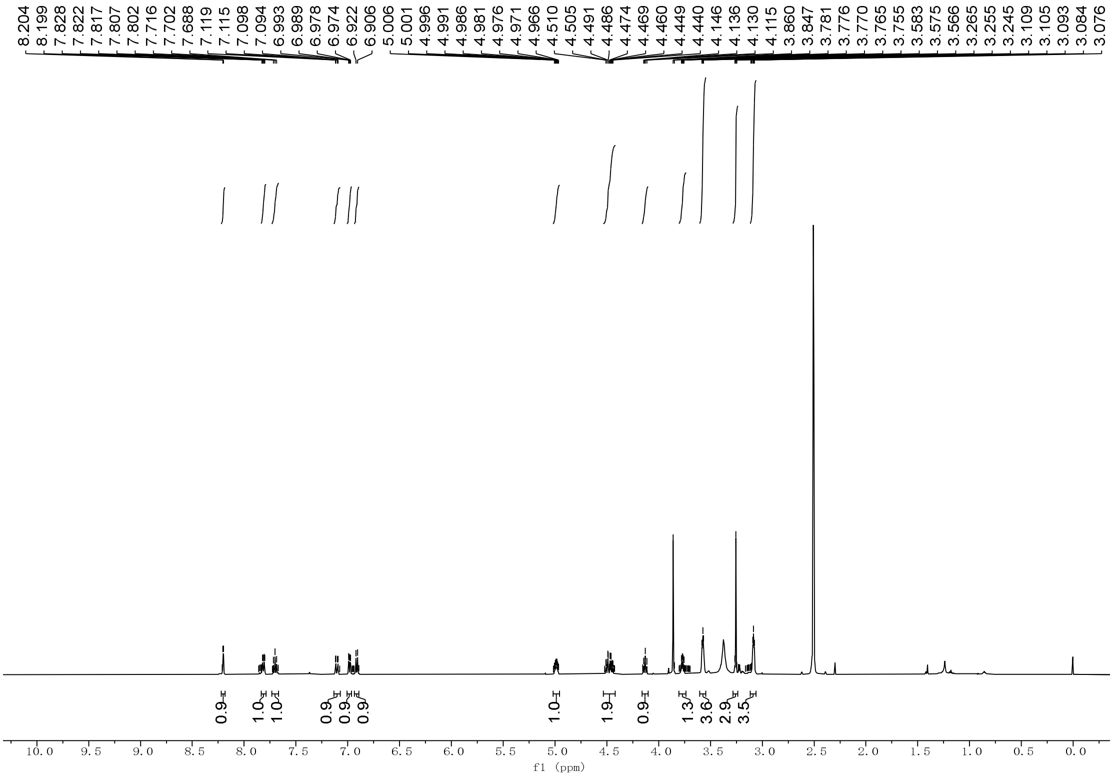

Figure S50. ^{1}H NMR Spectrum (DMSO-d_6, 600 MHz) of **12h**.

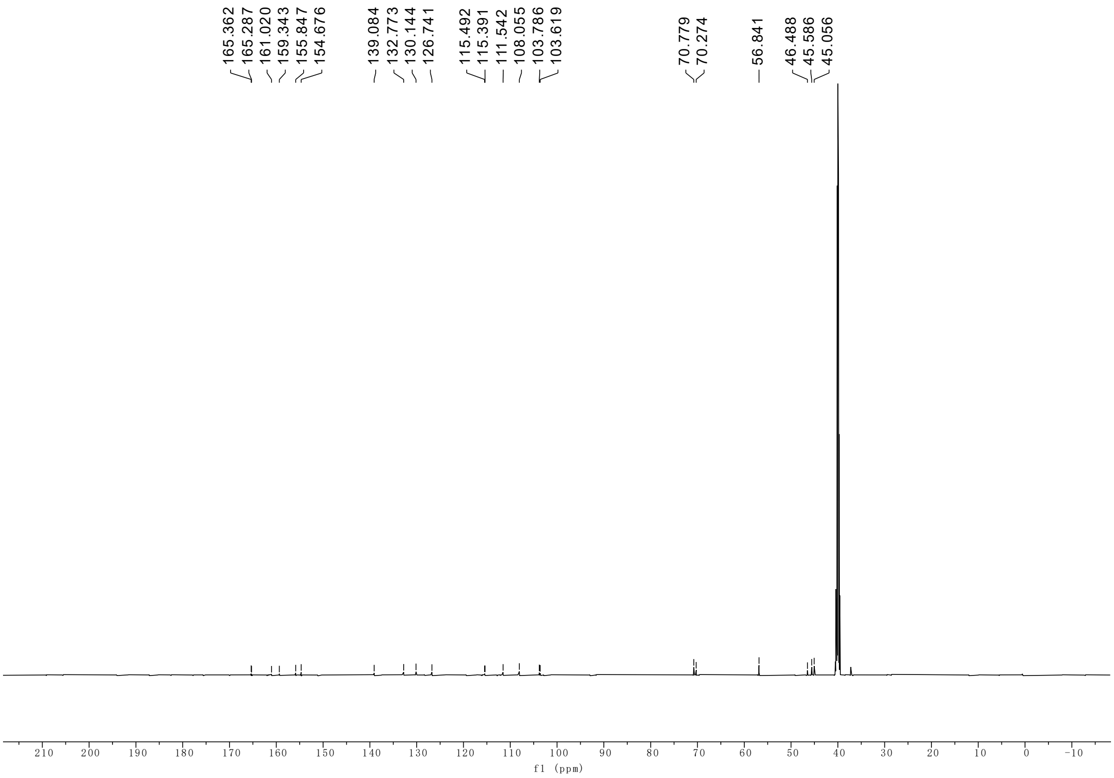

Figure S51. ^{13}C NMR Spectrum (DMSO-d_6, 151 MHz) of **12h**.

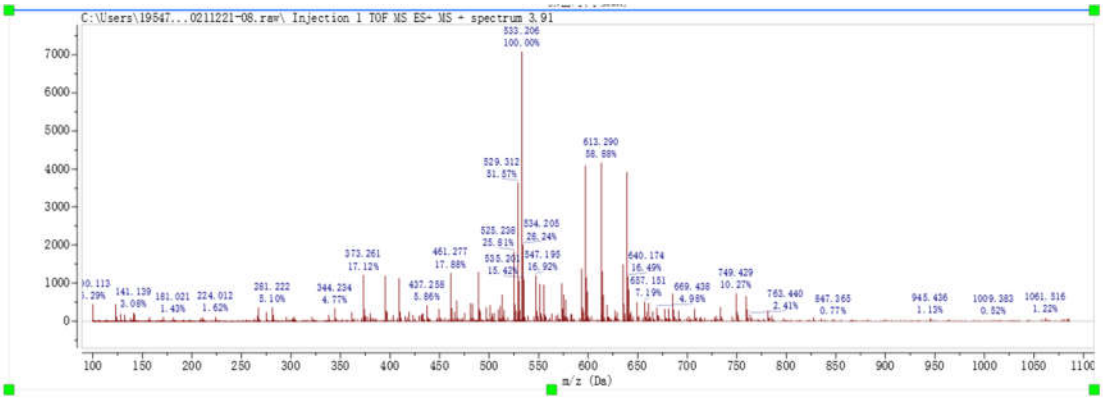

Figure S52. ES-MS for compound **12h.**
